# An Introduction to Generative Artificial Intelligence for Academics

**DOI:** 10.12688/f1000research.166513.1

**Published:** 2025-07-04

**Authors:** Nate Breznau, Hung H.V. Nguyen

**Affiliations:** 1 Organization and Program Planning, German Institute for Adult Education - Leibniz Center for Lifelong Learning, Bonn, NRW, 53175, Germany

**Keywords:** Gen AI, Artificial Neural Network, Large Language Models, LLMs, Generative Artificial Intelligence

## Abstract

General overview of artificial intelligence (AI) designed for academic students, workers, researchers, and teachers. A less technical introduction for those not familiar with computer science. It focuses primarily on generative AI (Gen AI), as this is the tool rapidly transforming every aspect of academic work. This primer covers four areas: 1. How does AI know what it knows? – An overview of artificial neural networks, large language models and “knowledge” ; 2. Ethics and best practices – Use cases, legal aspects and oversight; 3. Sources and tools - Support for the research process; 4. Prompting - Strategies to optimize interaction with Gen AI.

## 1. Introduction

This study aims to equip workers across academic roles with a foundational understanding of Artificial Intelligence (AI). The use of AI is already a major part of academic research; for example, machine learning and agent-based modeling methods exploded in the last decade. Since the 2020s, Generative AI (Gen AI) is publicly available and gaining popularity across all aspects of academia. This is a specific form of AI capable of producing its own unique and coherent outputs. Programs such as ChatGPT, Gemini, Copilot, and Meta-AI can assist with tasks like writing e-mails, bookkeeping, presentation preparation, data analysis, literature searches, and writing papers. Leveraging these tools effectively requires understanding how they work, using them responsibly, knowing what options exist, and how to best communicate with them through *prompting.*

This paper is semi-informal and follows the structure of a talk or course. It includes a mix of sources from both inside and outside academia. We, the authors, are practicing social and behavioral scientists, not computer scientists. We only grasp some of the technical aspects of Gen AI. Nonetheless, we find it imperative that people like us understand as much as possible to employ best practices when incorporating AI into our workflows and teaching. This is crucially important because AI is a General Purpose Technology ^a^ (GPT). Like electricity and the Internet, it will soon be used in all aspects of life, in and outside academia.

For starters, there is a broad range of technical jargon surrounding AI, therefore we compiled a glossary of terms accessible in our Online Appendix ^b^.

### 1.1 How does an AI know what it knows?

The ability of all mainstream Gen AI to respond to questions and commands derives from *artificial neural networks* (ANNs). These computing systems are modeled loosely after the neural structure of the human brain ^1^. Modern versions of these networks that power Gen AI store several billion parameters and require huge supercomputers to run computations with their networks of algorithms. However, if a user has sufficient storage and computing power, they can be copied, downloaded, and deployed locally, albeit usually in smaller versions. The saved parameters are activated each time the network is given an input. These parameters collectively and algorithmically generate a response to the input.

Gen AI’s “knowledge” comes from detecting patterns in vast amounts of data during a training process and developing prediction parameters based on those patterns. This means that it engages in pattern recognition rather than true comprehension or sentience.

### 1.2 Neural networks and learning from data

All ANNs consist of layers of interconnected neurons (nodes), which adjust the strength of their connections (weights) as they learn ^c^. This design is inspired by biological neurons, albeit at a much simpler level. The entire network is activated by an input; however, depending on the parameters and settings, not all of the network is activated to influence the output. Upon its construction, an ANN is neutral. It cannot predict anything meaningful. Without any training, the likelihood of the network generating any word (or image) is approximately equal. Through training, these likelihoods change – a process we as humans understand as the AI “learning.”

**Figure 1.**
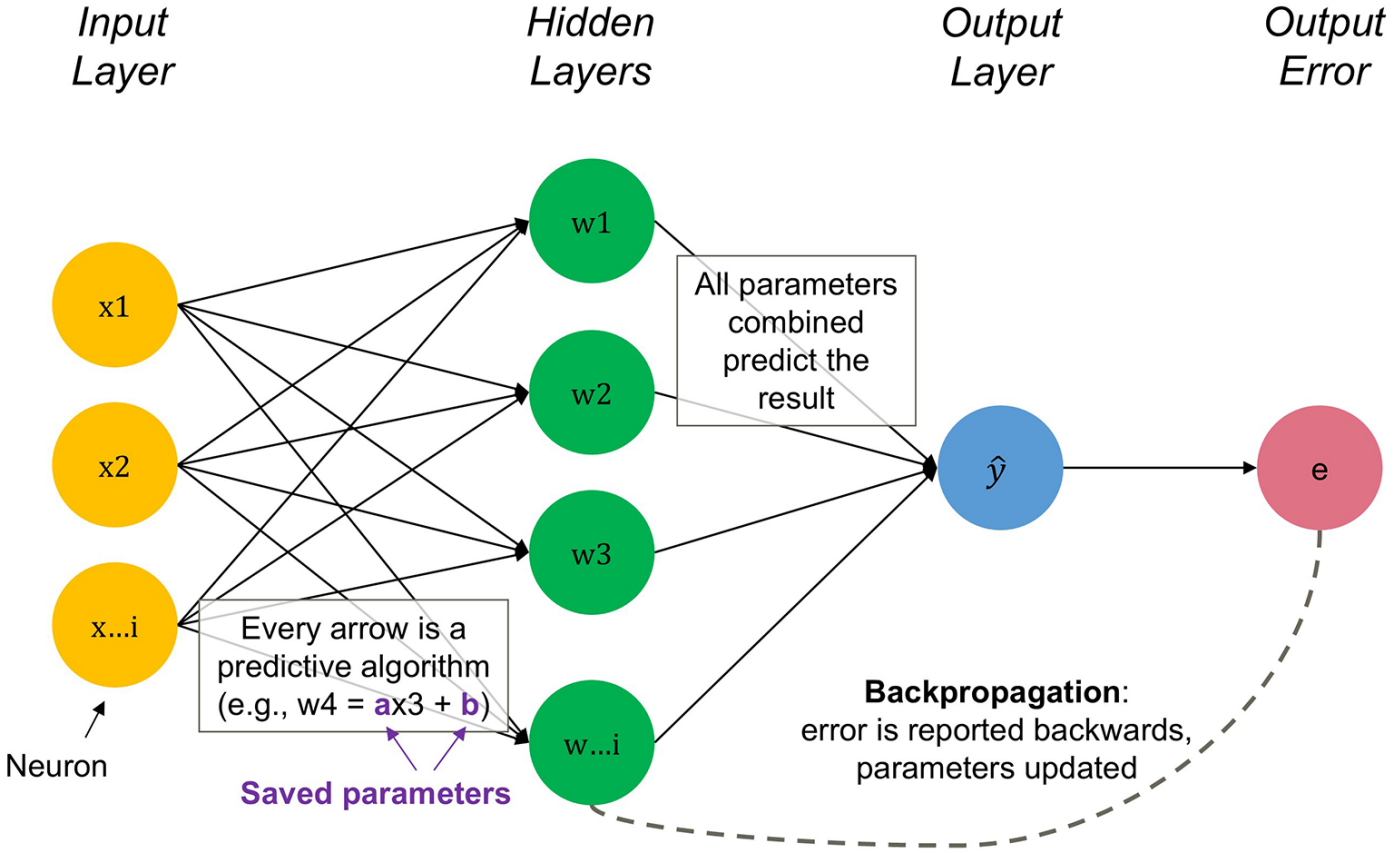
Visualization of an artificial neural network. Simplification of network and its embedded algorithms. Figure A1 shows the backpropagation process in more detail, see the Online Appendix ^p^. Source: Authors.

Learning works because each output generated during training is a certain distance from the correct answer. Distance by definition is the difference between the output and the correct answer; thus, it is the error which is used to adjust the strength of the neural connections, and the process is repeated until the distance approaches zero error, and the machine has the correct answer (or as close as possible). This is an algorithmic process known as *backpropagation.* It works by calculating errors in the network output and propagating those errors backward to adjust the weights ( *parameters*) on those neural linkages, nudging them in a direction that reduces the error ^2^. Through many iterations, a network “learns” to accurately map inputs to outputs. The weights of the neural connections are similar to coefficients in a regression model, which are stylized by the purple parameters in Figure 1 representing unique features of each node.

An intuitive way to think about AI learning is that the system uses iterations to gradually form a mathematical representation of concepts from the training data. In the case of image recognition: early layers of a neural network might detect low-level features (edges, colors), while deeper layers detect higher-level features (shapes, objects like “ears” or “whiskers”). A *layer* is a group of nodes, each with different levels of abstraction in the internal prediction process of the network. One layer passes information to the ‘next’ layer, going from the most abstract to the most specific in refining the prediction ^d^. The network does not understand “cats” or “dogs,” or even “ears” or “whiskers” like a human does; rather, it has tuned millions (or billions) of parameters such that certain patterns of pixel values will reliably lead it to output “cat” versus “dog.” Again, “reliably” refers to backpropagation learning to minimize errors.

Gen AI’s ability to appear knowledgeable is a direct function of the data it has seen. If trained on a large database of images or corpora of text, it internalizes an enormous range of facts and associations, such as in a human brain’s neural network. An artificial neural network does not have an active understanding or fact-checking mechanism beyond the patterns learned in the data. As a result, AI knowledge is inherently statistical; it knows which words or features tend to go together in the training data, not whether statements are objectively true. This has implications discussed later, such as the risk of confidently stated but false outputs. When Gen AI outputs are very far from the truth or the target of the prompt, they are known as *hallucinations.* We do not know exactly how a Gen AI hallucinates, but with millions of mathematical equations activated simultaneously, it is perhaps not surprising.

### 1.3 Large language models and knowledge representation

Artificial neural networks were developed in 1958 by Rosenblatt ^3^. It is the introduction of Large Language Models (LLMs) and Transformer architecture specifically since 2017 ^4^ which enables a neural network to become a Gen AI capable of written and spoken language communications, many of which can pass the Turing test today. This means that a user cannot tell if they are chatting with a human or machine in many cases ^5^. Artificial neural networks can work with images or other data, such as gene sequences. Therefore, an artificial neural network is not necessarily an LLM, but all LLMs today are artificial neural networks.

Most academics work with text, including numbers, written reports, emails, datasets, scientific papers, and spoken communication (which can be converted into text). It is easier to think about “text” for most of us, but LLMs use “tokens” instead of words as we know them. A *token* is a chunk of text. This could be a word or part of a word or punctuation. For example, the sentence “Generative AI is powerful” might be split into five tokens: “Gener”, “ative”, “AI”, “is”, “powerful” (note that the spaces before the tokens are usually part of the tokens and the learning process). The model does not understand whole words or sentences in the way humans do; instead, it learns and predicts the sequences of these tokens based on probability. In natural language processing, tokens provide basic data at a level below full words, that sometimes includes complete words, for measuring meaning in communication. Tokens allow languages to be processed much better than words. This means that when the model generates text, it chooses the most likely next token at each step, rather than full words or ideas. Stringing together the ‘next best’ token, output appears very coherent. In the example above “Gener” is a single token because it is a root of a word and could be combined with “-ate,” “-ating,” “-ations” and etc, and the predictive powers of the artificial neural network decide which next token is most likely, which ‘fits best.’

After training, a model is fed text that is converted into tokens and then passed through multiple layers of the neural network, each using an *attention mechanism* whereby the model dynamically weighs and focuses on the most relevant parts of the input text (tokens) in context. This allows the model to capture the relationships between words in a sentence or paragraph, even over long distances of text. Thus, it is not just predicting the next word, but also each next word in the context of all other words before it. Ultimately, the model uses this contextual information to generate a probability distribution over the next possible words, selecting the most likely continuation based on the learned patterns of language. However, this knowledge is not infallible. The model has no ground truth verification; it can only draw on the learned patterns.

Since the advent of the Transformer architecture, modern LLMs can be categorized into three types based on how they process the information ^6^. There are encoder-only, decoder-only, and encoder-decoder methods for this purpose. Those working with machine learning and text-as-data have likely heard of “BERT,” this is an encoder (literally: Bidirectional Encoder Representations from Transformers) ^7^. BERT was trained to understand contextual meanings using masked language modeling. For example, given the input “The [?] chased the cat”. It can predict “dog.” This requires bidirectional attention to all the information before and after the masked portion of the text. It uses full input to infer what is missing. It is not designed to generate coherent output sequences independently.

Unlike BERT, all Gen AI are decoders. OpenAI coined the term “GPT” to indicate a “Generative Pre-trained Transformer”. We are unsure if “ChatGPT” will become like “Google” is to refer to Internet searches, but it seems likely. A decoder learns to predict the next word in a sentence given the previous words. By training everything from books to websites, the model develops an internal representation of grammar, facts, and even common sense reasoning patterns to produce text in a human-like manner ^e^. Today when we say “LLM” in the context of Gen AI we almost always mean a decoder-only model like GPT.

A “GPT,” including any pre-trained Transformer model like GPT-4o, LLaMA or DeepSeek, is often termed a *foundation model* because it serves as a base that can perform many tasks when given appropriate prompts ^8^. They are multilingual, including all languages available for training, and include computer coding languages such as HTML, Python, and R. They can generate formulas, tables, and other complex formatting texts based on the tokens. For example, an LLM might complete “The capital of Bangladesh is ___” with “Dhaka” because during training, it encountered many sentences linking “Bangladesh” and “Dhaka”. Essentially, LLM knows this probabilistically because these words tend to appear together. We think of this in a sense that in any text, anywhere in the world in any language, Dhaka will be the capital of Bangladesh, and therefore a well-trained model would “know” it with a high probability.

Gen AI is generally a fine-tuned LLM. Fine-tuning takes a foundation model, and tunes it to become much more capable. A foundational model can only handle a limited amount of text at a time and has a narrower range of output in terms of length and style. Those who have tried to work with OpenAI’s API for example or used one of these pre-trained foundation models will know that the quality and coherence of the output is lower (more error). Fine-tuning changes this, expanding the possibilities. Fine-tuning also creates human-like properties of the model, a strategy developed initially for ChatBots to provide help services in lieu of human call center workers.

What is also added on top of a fine-tuned LLM that makes it a Gen AI, like those we interact with today, are *tools.* Tools allow it to process more than just text. For example, when we ask ChatGPT to generate an image, it switches from LLM to an image-generation ANN called DALL-E. It is trained to generate images from text (and image) inputs. Other tools Gen AI uses are, for example, browsing the Internet, running an analysis using specific software, and generating audio. These new tools can cause bizarre responses; for example, if they do not find very useful Internet sites, the Gen AI output is simply a product of the quality of these sites rather than an indicator of low quality training.

### 1.4 What Gen AI can and cannot do

Gen AI can be capable and helpful, but their results should always be fully vetted by humans. Human users are ultimately responsible for everything that they generate. Because every prediction is based on probability, the output comes with error, e.g., uncertainty and unreliability. Even without hallucinations, small disturbances to the ‘truth’ or whatever we as humans have that count as facts occurs. If asked about something obscure or not well represented in its training data, it might make an educated guess that sounds reasonable but is wrong. Early in its history, ChatGPT reported that *Strawberry has two r’s,* a trivial error, but illustrative of how AI does not truly understand spelling; it just makes the best guess in word-based output. A notoriously clear example of why humans need to vet everything is when an attorney submitted a legal brief in a court case that was written by ChatGPT. It passed the Turing test at first, but later a careful vetting revealed that it cited entirely nonexistent cases with AI-invented court opinions complete with fake citations that all sounded plausible and looked genuine ^9^.

The knowledge of an AI model is bounded by its training cutoff. If an LLM is trained on data up to 2023, it will not “know” news or facts that occurred after that. Tools can help Gen AI get around this because they can perform Internet searches and summarize newer events. In fact, via internet search and other tools the future of Gen AI will be in AI Agents rather than single ANNs.

An *AI Agent* extends beyond basic Gen AI to have a central AI that assigns tasks to other AIs via tools and actions that allow it to interact dynamically with the external environment. Common tools include web browsing, application programming interface (API) queries, image generation, and code execution. While a Gen AI model such as GPT can generate text based on pre-trained knowledge, an AI Agent can augment this ability by actively retrieving up-to-date information, performing tasks, and making decisions based on real-time inputs which it then uses to re-generate ‘better’ or more relevant answers iteratively.

Although most Gen AI models are technically agents because of their web search and code-running capabilities, we argue that the classification of an AI Agent should be based on having multiple artificial neural networks working in tandem, with one of them being a control center which reads the outputs of the others and then prompts them to change or alter their responses. With AI Agents, the ability to perform complex tasks iteratively with a centralized planning system makes them not only capable of logical problem solving, but also of doing what would take many humans a great deal of time to accomplish in just seconds or minutes. The difference between a Gen AI with tools and an AI Agent is the difference between the ChatGPT normal mode and ChatGPT ‘Deep Research’ mode. Deep Research was released by ChatGPT in 2023 with a paid subscription and, more recently, in Gemini 2025 with limited free public usage. These models can be used to develop research plans, iterative reasoning, and goal completion ^10^.

Another cunning and often hidden aspect of AI is bias. This means its errors are not always randomly distributed. AI models learn from historical data and thus pick up and even amplify societal biases present in the data, which could include prejudices against various social groups ^11, 12^. For instance, image-generating AI might consistently depict certain professions or roles with specific genders or ethnicities owing to skewed training data, or a language model might produce biased statements about marginalized groups based on data that are part of the structures that marginalize those groups in the first place ^13^. Most academic institutions’ goals are to produce science as something democratic and following national and local laws ensuring fairness, thus addressing bias in AI outputs is an ongoing challenge across all academic jobs.

Some companies fine-tune strong reactions into their models to inputs that sounds prejudiced or insensitive. For example, we prompted Google’s Gemini asking it to produce an image of a ‘light-skinned woman’ which it declined for reasons of potential societal harm. The utility of such a response to address social inequality is marginal at best. Perhaps even more odd is that it went on to generate a dark-skinned man instead. This showed randomness in which marginalized groups it ‘safeguarded’. This exercise shows that Gen AI itself is not yet competent at detecting or accurately discerning racist or biased usage. In higher profile cases, the Gen AI Grok produced output denying the Holocaust ^14^ and Gemini could easily be tricked into storing malicious content which would impact its later outputs, thus opening the door for any malicious or biased output intentions.

Strictly speaking, AI and therefore AI Agents do not have conscious knowledge of anything and cannot, therefore, have values or understand the meaning of communication; this claim is known as the *neutrality thesis.* The problem is that the results of Gen AI outputs contradict the neutrality thesis when taken literally, which can create confusion for users. Gen AI has a deep statistical understanding of meaning in language, and values themselves are embedded in linguistic meaning; therefore, it does produce, or at least reproduce, values. In many tasks AI ‘reasoning’ and creative thinking already surpasses humans ^15^. Some argue that knowledge and values cannot exist independently of one another, so if we accept that Gen AI has knowledge we inferentially admit it has values ^16– 18^. We do not take a strong position here, as this would require a better understanding of what is occurring in an artificial neural network and a human brain neural network. What we want to be very clear about is that Gen AI will provide value-laden communications depending on the input, so it *expresses* values, and this should be taken into consideration.

AI systems “know” what they know through pattern learning on large datasets using neural networks. This gives them remarkable pattern-matching abilities but not common sense or reliable judgment in the way humans possess. They are biased in a way that is totally different from how a human might be biased. It is fair to imagine that every Gen AI response could lie somewhere on a distribution from complete nonsense to watertight logical statement, which means that we need to be perpetually mindful and critically vet what we get from it.

## 2. Ethics and best practices

Although AI offers powerful capabilities, its use in research raises ethical and practical concerns. In 1965, science fiction writer Stanisław Lem, imagined a universe in which highly advanced, sentient machines behaved in unexpected and manipulative, ways. His character Trurl in The Cyberiad offers a practical warning, relevant today: “Do not trust the machine. It lies. It tells you what you want to hear.” Whether machines become sentient or not, human desires can be exploited by Gen AI because it is trained on them. Because researchers seek publications and status, we believe Gen AI can and will lead to questionable research practices. Gen AI can generate falsified factual sources, human-like but fake survey responses and p-hack on a scale beyond what most single humans or research teams are capable of. This poses challenges for researchers, funding institutions, public trust and science information specialists (e.g., in libraries).

### 2.1 The “All-in-One” AI myth and tool diversity

There is a temptation, if not belief, among users that Gen AI is a magic solution for everything: a proverbial *eierlegende Wollmilchsau* (an egg-laying milk pig!) ^f^. Currently and certainly into the near future, this is a false and dangerous belief. AI tools have different purposes that support but do not replace traditional tools for academic work. An Internet search function for AI, for example, relies on up-to-date and useful information on the Internet which can only be generated by human knowledge. Gen AI can brainstorm or draft text, but a specialized finder tool might be better for literature or systematic reviews, and conventional statistical software remains necessary for data analysis. Even if an AI can run statistical analyses, it might not be doing what users think, or could hallucinate results altogether.

Using AI appropriately means not forcing it into roles it is not trained for. Imagine a social science researcher using a Gen AI model such as ChatGPT to conduct qualitative coding of interview transcripts, expecting it to reliably identify “power dynamics” and “emotional labor”. These terms are highly specific, may require specialized knowledge if not new definitions on the part of a researcher, and are unlikely to be a major part of training a general-purpose Gen AI. A better approach would be to train a model (such as BERT) to identify these issues based on specific and highly complex qualitative parameters defined by a researcher.

Ideally AI is complementary. It augments academic work, and can only do so with careful attention to its risks, realistic uses and biases. There is enormous variety and simultaneously a lack of transparency in AI tools, which goes against the ideal of reproducible research as good scientific practice. Artificial neural networks have so many nodes and layers that we do not really know why they obtain the answers they obtain, other than by refining parameters ^19^. This is why identifying causes within ANNs is like trying to identify causality in the human brain. This is far too complex and we lack the ability to measure it in real time.

### 2.2 Human oversight and critical evaluation

When AI generates text or analysis, the researcher must evaluate the work in the same manner as if they had done it themselves. Gen AI is more akin to an assistant who can draft or summarize content, which needs to be checked by the supervisor responsible. If one uses AI to help write a section of a paper, the onus is on the human author to ensure that the content is accurate and includes accurate and real citations for any fact. *We emphatically implore researchers to never use Gen AI generated text for any work they produce.* Having outlines or text generated as examples can be extremely helpful but should be entirely (re-)written by the researcher(s). Copying and pasting from Gen AI text should never be done for work prepared for a public audience in our opinion.

Maintaining transparency is ethical and follows ideal open science practices ^20, 21^. Many journals and institutions now have policies on AI-assisted writing, which typically require disclosure if AI is used to generate content. For instance, *Nature* journals in 2023 stated that AI cannot be listed as an author, and any use in producing a manuscript must be disclosed to readers ^22^. The National Institutes of Health, German Research Foundation (DFG ^g^) Code of Good Scientific Practice and Leibniz Association’s guidelines emphasize honesty and transparency in acknowledging the use of generative models in research ^23– 25^. Even in the absence of formal guidelines, it follows from academic integrity that the role of AI must be stated in scientific research. Responsibility does not lie in software, developer, or provider in any research context. The researcher remains responsible, and AI is no exception.

The topic of plagiarism and authorship is complicated by Gen AI because it can produce text that resembles or is partially identical to existing sources given the right prompt. If ChatGPT summarizes a paper, one should still cite that original paper, not just say “ChatGPT said …” This is a similar best practice recommendation as with Wikipedia. It is best to track down actual sources. Gen AI is highly trained on Wikipedia, but the exact training data is not transparent; therefore, the workflow to produce it is an opaque box.

Additionally, AI output itself is not copyrightable under current law because there is no human creativity involved; it is not intellectual property ^26^. This means that researchers cannot claim AI-written texts or AI-created images as their own copyrighted work. At the same time, they do not need permission to use AI-generated texts or images, although using them without disclosure may breach ethics or law. Nonetheless, if a human substantially edits or curates the AI output, human-modified content may be eligible for copyright; the legal landscape is evolving, and it is difficult to anticipate best practices when so many areas of AI are still not covered by various national laws.

It is also difficult to anticipate the potential corruption of the data faced by scientists. A recent study revealed that over a third of survey respondents used Gen AI to help them answer open-ended questions ^27^. This means that some survey data are corrupted and no longer representative of human attitudes.

### 2.3 Use and misuse

Because AI can inadvertently reinforce stereotypes or give misleading impressions owing to biases in the training data, responsible use involves awareness of these biases and active efforts to mitigate them. When using AI to teach others, public relations, or to promote research, it is important to review outputs for any inappropriate or biased content that could negatively influence the impression of the audience. As previously discussed, it is easy to use AI to support racist, sexist, or otherwise oppressive agendas inadvertently but also intentionally. This is a real danger, especially because the content that future AI will be trained on is now being produced by current AI.

Generative AI can create very realistic fake content, raising concerns about misinformation. For example, it can mimic famous people and politicians’ voices, making it easy to spread propaganda ^28^. This is compounded by video-generating-AI which for a few months now has produced content that superficially cannot be distinguished from real video ^29^. Generating deep fake content and selling it as science is academic fraud. Less malicious, but still problematic, is over-reliance on AI, such that it diminishes the researcher’s own critical engagement. Imagine students cheating to beat the tests they took, who become reliant on their sophisticated cheating skills to maintain future success. This is sometimes called *cognitive offloading* ^30, 31^.

Cognitive offloading is not necessarily bad, for example researchers can give more menial repetitive tasks to AI to support their work. The problem is that if too much is offloaded, researchers may end up offloading their own knowledge acquisition process. A researcher could theoretically become widely cited in a field in which they personally know very little through the use of AI. There is a risk that AI’s capacity to construct logical arguments, engage in effective literature reviews, or even come up with research questions may collectively denigrate academic skills because they are overly offloaded ^32^. Simultaneously, AI can improve our research and demand idiosyncratic skill development to achieve the best outcomes. Scientific work using AI requires a balance between effort and return. To achieve efficiency gains, an investment in time is first required to build AI expertise.

For over a decade, academics have used Google searches and Wikipedia articles to extract diverse information to summarize and use in their own writing. AI is not fundamentally different; it can summarize much faster and more comprehensively in most cases. Again, this is why we think simply never copying and pasting AI-generated text into a paper, but instead using it as a basis to write something is the best practice with Gen AI. When it is self-written, there is no reason to cite AI as having generated text. In fairness, this is a grey area, as it might be appropriate to mention that AI helped. However, consider this: we are not reporting in our papers when we used Google or Wikipedia, and question whether referring to Gen AI is fundamentally different.

Educators and institutions are grappling with guidelines and codes, and developing AI detection tools such as originality.ai and GPTZero ^33^. Fundamentally, responsibility lies in individuals upholding academic integrity. 

The grey area is complicated by two opposing directions of trust: On the one hand, it seems that people trust the responses of Gen-AI. In fact, in a recent study, they trusted it more than human lawyers ^34^. On the other hand, we (researchers) are skeptical of others who generate work using AI. For example, a recent study showed that scholars trust others’ work less when they know it is generated with support from AI ^35^. This creates an incentive to use AI without reporting it, a potentially perverse incentive.

The ideal AI is used to augment human skills and cognition, and not to replace them. Researchers still must cultivate critical reading, writing, and analysis abilities. AI is a support, not a substitute.

### 2.4 Using Gen AI locally

Most of us use Gen AI through an app that we access via the Internet. In doing so, we access the most powerful versions of Gen AI because they were developed by companies that invested incredible amounts of money and time into training them with gargantuan computer systems allowing millions of simultaneous users to query them. Some companies share their models with the public open source in the interest of science and shared technological development. This means users can download and run the models *locally.* Running a model locally means that the entire ANN is saved on the device and can generate output without contact or resources from a server ^36^.

A local model is attractive, because users can provide it with anything they want, including sensitive data. For example, many workplaces have folders containing long documents that provide information on rules, rights, contacts, contracts, persons, and procedures. This can be incredibly time-consuming to navigate. It would be unethical, if not illegal, to upload workplace-sensitive information into Internet-based AI. With a local model, it is possible to search, summarize or analyze (private, sensitive) text as an input and find answers without violating privacy laws or ethical guidelines. A local LLM that is not fine-tuned can only handle a limited amount of input. It would need to be fine-tuned (further trained) to develop knowledge of the local folder and/or documents to be truly effective. This fine-tuning is very likely, beyond the skills or comfort zones of most academics ^h^.

Privacy when using Gen AI is a perpetual concern. There are usage agreements that purportedly protect the user. For example, nearly all paid subscription Gen AI versions that we are aware of come with the option to “never” store prompts or allow them to be used for training. Microsoft claims that Copilot conforms to all the ethical and legal guidelines agreed upon in user agreements, making it an attractive option for companies that already use a suite of Microsoft tools. There are also current discussions about making AI prompt storage mandatory and security concerns with AI from other countries being used for espionage.

## 3. Sources and tools

AI can assist researchers across *all* phases of their scholarly work. There are so many tools available that we cannot review all, nor predict which will be adopted and which will fade. Here, we provide a quick look at some already being widely adopted for research, writing, and publishing processes. ^37^

### 3.1 General-purpose generative AI tools for research support

Several general-purpose Generative AI tools have emerged in the evolving landscape of academic research, offering broad applications that assist researchers across multiple stages in their workflow. These tools are not confined to specific tasks, but provide versatile support, enhancing productivity and efficiency in research activities. Most have or are developing AI agent capabilities for deep research. See Table 1, last updated May 16 ^th^, 2025.

**Table 1.** Some common general purpose Gen AI and AI agents ^q^.

**ChatGPT (OpenAI, USA).** OpenAI’s ChatGPT, particularly its GPT-4 model, has become a widely adopted tool in academic settings. Its capabilities include drafting and editing text, summarizing literature, generating code snippets, and answering domain-specific questions. Its adaptability makes it suitable for various tasks, from brainstorming research ideas to refining manuscripts. The free version of ChatGPT gives access to GPT-3.5, while GPT-4 (more specifically, GPT-4-turbo) is available via a paid plan. It has an AI Agent ‘Deep Research’, the first of its kind but only for paid subscribers.
**DeepSeek AI (China).** DeepSeek AI is designed with a focus on data analysis and research applications. It excels in extracting insights from large datasets and generating detailed reports, making it valuable for researchers and data analysts engaged in complex data processing tasks. The free version offers access to both DeepSeek-V2 and DeepSeek-Coder with strong capabilities in multilingual code generation and natural language understanding. As of 2024, agent capabilities such as document querying and code execution are in development but not yet fully deployed.
**Gemini (Google, USA).** Google’s Gemini AI model is recognized for its creativity and multimedia content generation capabilities. It supports tasks such as content creation, language translation, and summarization, providing researchers with tools to enhance their communication and dissemination of findings. Gemini is freely available via Google’s Bard interface (rebranded as Gemini), with access to Gemini 1.5 Pro in the Pro tier. Gemini recently made its Deep Research free without a paid plan for a limited number of uses per month. We have not confirmed this, but because it is integrated into the Google infrastructure, its internet search capabilities should be higher quality and faster than any other AI Agent.
**Mistral AI (France).** Mistral AI is a French AI company developing open-weight large language models, including **Mistral 7B** and **Mixtral**, with a focus on transparency, efficiency, and multilingual capability. These models are freely available and designed to support broad use cases such as text generation, summarization, coding, and translation—offering researchers high performance without platform lock-in. While there is no official web-based interface from Mistral AI itself, their models are integrated into free platforms like HuggingChat and Le Chat, where users can access basic capabilities. Mistral models are being deployed in early-stage agent frameworks, but as of now, they do not natively support AI Agents.
**Perplexity AI (USA).** Perplexity AI functions as an AI-powered search engine, offering real-time information retrieval with cited sources. Its strength lies in providing fact-based research support, enabling researchers to access accurate and up-to-date information efficiently. The free version allows unlimited question-answering with real-time search and reference links, though GPT-4-based responses are limited to Pro users. Perplexity’s “Pro Search” behaves like an AI Agent by conducting multi-step retrievals and comparisons across sources.
**Microsoft Copilot (USA).** Integrated into the Microsoft 365 suite, Copilot serves as a comprehensive assistant for researchers. It aids in drafting documents, analyzing data, and summarizing information within familiar applications like Word and Excel. Its seamless integration into existing workflows enhances its utility in academic research. Copilot is available in limited form through free Microsoft accounts (e.g., Edge Copilot), while full integration with Office apps requires enterprise or subscription access. Microsoft is also incorporating agent-like features into Copilot, including goal-oriented assistance, plug-ins, and integration with external APIs, but is not yet a full AI Agent.
**Qwen (Alibaba, China).** Advanced Gen AI applications to compete with all other Gen AI products. It is particularly good for developing and debugging code, but can generate images and video, it offers a very large free version that can handle more tokens than most. It also offers AI Agent capabilities.

These general-purpose Gen AI tools offer researchers a range of functionalities that streamline various aspects of the research process. While they provide broad support, it is essential to recognize that their effectiveness may vary depending on the specificity and complexity of the tasks at hand. Researchers should consider the unique features and strengths of each tool to select the most appropriate one according to their needs. Given that they all offer different responses to prompting, it could be useful to ask several the same question and then extract manually the most useful points.

### 3.2 Literature reviews: Finding, connecting, summarizing

Researchers traditionally follow certain steps when conducting literature reviews or exploratory research on a topic: formulating search queries (keywords, subject headings), using databases or search engines, scanning titles/abstracts for relevance, reading papers, and so on. AI augments these steps by allowing users to interact more naturally with information and automate some of their grunt work.

For example, *natural language querying* is now possible; instead of coming up with a *Boolean* search string (which requires “and” and “or” and “not” operators), a researcher can ask in plain language things like: “What are recent findings on the impact of early childhood education on literacy rates, please report results only from psychology and economics”. Such questions are ideal for a model with an Internet search tool, which greatly reduces the risk of references being (slightly) incorrect. This contrasts with the classical approach of trying multiple keyword combinations in databases, such as Google Scholar, ERIC, and PsycInfo.

Specialized AI literature review models are also available. These essentially perform two tasks that are more specialized than general Gen AI. There are *finders* that are AI-powered search engines fine-tuned to find relevant academic studies and documents given a search query. They typically use large academic databases and language models to interpret queries and summarize the results. They help answer questions like “What are the main findings about X?” by retrieving papers and summarizing the findings.

Here is a non-exhaustive list of tools to which we are familiar.

**Semantic Scholar:** Perhaps the most well-known AI-powered search tool for academic literature. The user interface is like that of Google Scholar. Google Scholar is algorithmically powered but is not yet AI driven, although it uses AI to better organize all the information behind the algorithmic search.

**Research Rabbit**: A search and citation network article finder *and* connector. Includes a plug-in for the open-source citation software Zotero.

**Consensus**: An AI-powered academic search that answers questions by pulling out snippets from papers. It focuses on peer-reviewed research and provides an evidence-based summary. Consensus works best for well-studied questions in large fields, such as medicine and psychology. It searches a large database (over 200 million papers) and highlights consensus or disagreement among studies.

**Elicit**: Built to find relevant papers, present key information such as abstracts, and extract specific data (e.g., sample size and methodologies) from PDFs using natural language queries. It is particularly aimed at helping with systematic reviews (finding evidence without needing perfect keywords) and prioritizing results by user specified criteria. Elicit’s database is built on Semantic Scholar and other sources, which means it covers many open-access articles and those with detailed abstracts. Like Consensus, it excels in finding English-language open literature, and it may be less effective for paywalled or non-English sources.

**ORKG Ask:** A tool of the Open Research Knowledge Graph project, which uses a knowledge graph and LLM for scientific search. It is designed to present the results in a comparative tabular format, showing the methods and findings across papers (useful for literature reviews). It draws on the CORE ^38^ database (which aggregates the open-access repository content). Its strength is that it provides a structured overview of research topics (e.g., comparing multiple studies’ results side-by-side).

**AI Agents (e.g., ChatGPT and Gemini)**: General AI Agents that use Deep Research can be highly effective finders. For instance, Microsoft’s new Bing Deep Search (which integrates GPT-4 with a web search) can retrieve sources from the Internet and even cite them, acting somewhat like an AI research assistant. However, general tools may lack domain-specific filtering, and they can return fewer scholarly sources if not carefully prompted.

**Figure 2.**
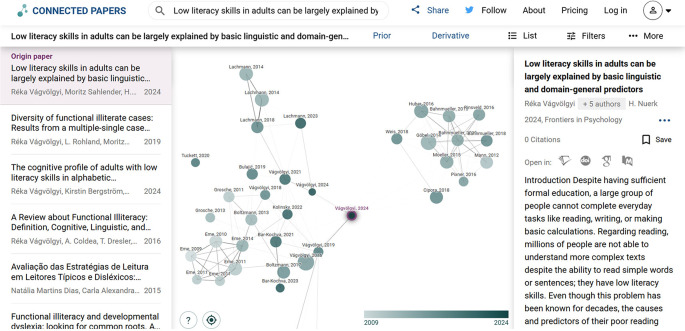
An AI “Connector” tool in action. Screenshot of a connected paper graph generated for the original paper (at the center). Each node is a paper, and the edges (lines) indicate connections based on citation patterns and topic similarity. Such visual tools help to identify clusters of research and pivotal works at a glance, complementing traditional searches.

Another class of models is *connectors* that explore the connections between known relevant papers. Instead of starting with a question, the researcher starts with one or more seed papers that they already deem important, and the tool finds related works through citation networks and semantic similarity. It works similarly to old-fashioned systematic reviews, which start by finding all cited and citation-containing papers to work with. Figure 2 demonstrates the visual output of a connector Gen AI.

Here are some examples:

**Connected Papers**: This tool creates a visual graph of papers related to a chosen origin paper. The graph nodes are papers, and proximity indicates similarity (often based on co-citation and bibliographic coupling analyses enhanced by NLP).

**Local Citation Network**: A tool that takes the DOI of a paper and builds a citation network of papers that cited it or that it cited. It is useful for tracing the lineage of an idea; one can see the ancestors and descendants of a research article. It supports switching between data sources, such as OpenAlex and CrossRef, and is privacy-focused (no login needed).

**Inciteful** (and **Research Rabbit**): This allows the interactive exploration of citation networks with more user control. For example, adding multiple papers to a collection and then seeing common citations or references.

**Traditional indexes with AI features**: Traditional academic platforms such as Web of Science or Scopus now integrate AI to suggest related articles beyond simple citation links using keywords or topics. 

**NotebooksLM**: This product is excellent for connecting and summarizing papers, but the user must first upload or link papers. It has many other features, such as the capacity to generate a podcast from one study or several studies and to allow chatting with a bunch of studies at once.

The benefit of connector tools is the capacity to find relevant research that keyword searches miss, because the terminology differs. Especially in interdisciplinary research, two papers might discuss similar concepts with different jargons; a connector tool that notices they cite a common third paper, for example, can bridge that gap for you.

New tools provide systematic review functions using AI (e.g., DistillerSR or LaserAI), but we are unaware of any of these that are free for the user, and we have not tested them. It is clear, however, that automated systematic reviews will soon become the norm; more, we cannot predict. ^39^

### 3.3 Analysis and data support

General-purpose Gen AI can perform powerful calculations and show its work in formulas and proofs. As math is precise, it is easy for AI to learn through error reduction. In our experience, Gen AI is nearly perfect when it comes to mathematical calculations ^i^ and very good in code generation for the most commonly used statistical software that are widely discussed on the Internet (e.g., Python and R are great, Stata and SPSS are not as good).

**Power Analysis**: In our combined experience, with decent prompting (see section 4), Gen-AI will make accurate calculations of the sample size, alpha, detectable effect size, and more, based on research design inputs. This generates the equations used to calculate the results.

**Data Extraction**: Gen AI often has access to most databases, such as the OECD or Eurostat, and can extract and prepare data. Data can be converted into almost any format for downloading. This is the case for ChatGPT and Gemini, and we assume that it works the most. Gen AI can also extract structured data from text. Although Gen AI output is not always reproducible given customizations, different computing environments, and proprietary architectures, it is possible to engineer prompts or ‘promptbooks’ in a way that they become more reliable via testing and error minimization and are relatively reproducible ^40^. Promptbooks serve as structured guides that catalog effective prompt patterns for various tasks. These codebooks compile tested prompt templates, strategies, and examples, enabling users to elicit desired outputs from AI models more efficiently. For example, unstructured descriptions of people in newspapers can be used to extract specific variables, such as age, sex, education, and occupation, using prompting (prompt book) techniques ^41^.

**Data Analysis:** We can upload data and ask Gen AI to execute the code to analyze the data. In this case, users should exercise caution. For example, ChatGPT can only run Python or R. Additionally, the user has little or no control over packages or versioning. The range of tasks generally includes everything Python and R can perform in the case of the former, which includes machine-learning applications. For example, Prandner et al. used ChatGPT to generate code and analyze data ^42^. They discovered that Gen AI excelled at many but not all tasks and was poignantly better with open source and highly discussed software on the Web like R in comparison to others such as SPSS.

**Annotation:** Researchers have had great success in uploading text and asking Gen AI to qualitatively analyze and ‘code’ the text (what is known today as annotation). There is evidence that Gen AI can annotate as well as human coders in most cases ^43^ and often better ^44, 45^, but sometimes there are exceptional biases and systematically distorted errors, as seen in an example of coding interviews with displaced Rohinga ^46^.

**Fine-tuning **: Researchers seeking to train a machine-learning algorithm can ask Gen-AI to generate new sentences based on existing sentences to increase the size of the training data. These sentences can maintain the same meaning from a natural language processing perspective but reduce Type I and Type II error rates (higher F1, precision, and recall).

**Replication**: AI is being used successfully to perform replications of previous studies. This still requires human operation. But a recent study demonstrated that human’s who followed all instructions given to them by a Gen AI replicator, were equally as successful as humans replicating without AI in reproducing others’ findings. ^47^

## 4. How to interact with Gen AI: Prompting

Most mainstream Gen AI contain tools such as web searches and API. Although there are differences in the training (the parameters of the ANN) and power (GPU and server capacities) of the different Gen AI, the real differences in performance come from the user. Gen AI is highly sensitive to how users phrase their input, which is known as *prompting.*

At its core, a prompt is simply the input or question you provide to the AI model. However, writing a prompt is like writing the instructions for a very literal-minded, knowledgeable, but non-sentient assistant: You often need to be specific and clear about what you want, often in ways you do not need to be with a human. We have developed these definitions to guide users.

**Prompt**: Structured input comprising text, code, or other data designed to elicit a specific output from a Generative Artificial Intelligence (Gen AI) model by framing the task, constraints, and context.

**Prompting**: The practice of formulating and refining prompts to guide the model towards the desired outputs. In short, prompting is how we “program” a model with instructions every time we use it, without changing the model code or parameters.

If you want AI to help summarize an article, a prompt might be: *“Summarize the following article in one paragraph:”* followed by the article text. A more elaborate prompt may set the context and tone: *“You are an expert in the field. Summarize the following article in one paragraph, focusing on the main findings and their significance, in a neutral academic tone:”* followed by the text.

We categorize best prompting practices in the following four sections: 4.1. What you want, 4.2. How you want it, 4.3. Tasks and examples, and 4.4. Interactive iteration.

### 4.1 What do you want?

Clearly specify the task that you want the AI to perform. The uncertainty expressed in a prompt is directly reflected as uncertainty in the AI answer. Simple prompts like, “describe social homophiliy” or “what is a regression” will lead to answers like what you would find on Wikipedia. Generalized, generic knowledge of the subject. If you tell the AI to “describe how social homophiliy impacts assortative mating in a country that has an ethnically diverse population and very high inequality,” the answer will be far more targeted and sophisticated. Assume that AI has nearly unlimited knowledge, but to tap into it, correct words must be used. Words and their ordering have meanings and linkages with other meanings.

Some common ideas of “what” to ask for are translation, summary, drafting (an email, report, social media post), comparing things (like articles or data), data analysis, internet search and interpretation, research design, filtering (removing unwanted things from text), reformatting (into certain styles), and rewriting (to have a more polite or academic tone). We are now in a completely different ‘ball game’ with Gen AI. Because AI is a trained artificial ‘brain,’ we could potentially get anything out of it, depending on the prompt.

With the help of Gen AI (here ChatGPT 4o), we came up with some research tasks that humans cannot do alone. An *auto-synthesized interdisciplinary literature map.* This could be the basis for an automated systematic literature review. This would most likely require an AI Agent, as the citations must be checked to ensure that they are not only accurate but also real. A *synthetic peer review panel* could prompt three different peer reviewers with different disciplines, expertise, and emotional states to critically review our paper. We could also ask AI to create *theoretical functions* that explain some phenomena in the world (see Online Appendix Prompt 1 ^j^ for an example of a likelihood of war function).

### 4.2 How do you want it?

An effective prompt includes details of the desired format, length, style, and other output criteria. Currently, almost all Gen AI can output office-style documents such as. docx, .pptx, images, .xlsx, .csv, .json, .md, .html and etc ^k^. For text, it can come in any style that a word processor can produce, for example, a list of bullet points, formulas, bold, italics, etc. The user can specify the style, for example, a certain academic style, such as the American Psychological Association, or in the style of a social media post or journalist. The font, spacing, and version of the language (German language in Swiss vs. German style) can all be specified. The tone can be adjusted and having the Gen AI ‘pretend’ it is a peer reviewer, critical boss, or emotionally sensitive colleague could all lead to more precise and useful outputs.

Specify the target audience if applicable. This will help an AI to adjust its complexity and use relevant information. For example, “Explain for a 12-year-old” versus “Provide a detailed technical explanation suitable for practicing researchers with at least a PhD in a social science discipline” would lead to very different outputs. For things that are really new or difficult for you, try asking “explain for a child,” and then adjusting the age upward.

*Temperature* is a Gen AI-specific setting that ranges from 0 to 1. A high temperature (e.g., 0.8) yields more creative and varied outputs. A low temperature (e.g., 0.2) yields more focused and deterministic outputs. Setting this value to zero should result in the highest (and dryest) amount of factually consistent information. Most Gen AI will understand “Temperature 0.5”, but if not, you can implicitly guide this through wording (for example: “Give a straightforward, literal answer” vs “Feel free to be imaginative”).

It is important to understand that *every word* and *word combination* counts in shaping the output from Gen AI. By clearly outlining how you want the answer, you reduce the chance of getting a wall of text when you want a table or an overly casual answer when you want mathematical proof. Our human tendency to ask for things in a polite manner using “please” and “thank you” for example matters sometimes a great deal and could actually lead to lower quality outputs ^l^. Gen AI will ‘think’ that it needs to formulate a polite response. Politeness from an NLP perspective means that it is acceptable to hide the truth somewhat to avoid upsetting the prompter. If you do not get what you want through prompting, think carefully about the word choice.

Most Gen AI have customization features. For example, in ChatGPT, one can click on the “Personalize” menu and set features that apply to every prompt. For example, one of us has as their settings the name the ChatGPT should call them, that they are an “Experienced scientific researcher” that the traits should be “Scientific, blunt, low temperature and using logic” and that they “use ChatGPT mostly for scientific writing, research and analysis.” This can save time, so a researcher does not always have to add these customizations to every prompt or chat.

### 4.3 Tasking with examples

At the core of a prompt is a *task* that a Gen AI should undertake. The ideal prompt contains a single task, and everything in the prompt is related to that single task. There are cases that require more than one task in a single prompt, and in these cases the tasks should be numbered or lettered, otherwise the different tasks ‘bleed’ into each other leading to mixed or undesired outcomes. It is beneficial to provide context, examples, or roles to get the most out of tasking.

Provide context. Gen AIs have no knowledge or memory of the world beyond what you give in the prompt, and their ANN is built on training data. If your question depends on a specific context (such as the passage of text, data, or the last thing you talked about in the conversation), make sure to include or reference it in the prompt. For example, instead of saying, ‘What does this result mean?’ (with no context), say “Given the results of the study (summarized as: XYZ), what does this result mean for practical applications in education?” Including a brief summary or the relevant excerpt in the prompt allows the model to base its answer on that rather than on general stored knowledge.

Provide examples. Examples are known as *shots* in computer science. You can sometimes save a lot of time trying to describe exactly what you want and how you want it by simply giving a few shots. You may want an abstract summarizing your work because you plan to submit it to a specific academic journal. You can give Gen AI a few abstracts from that journal and ask it to craft your abstract following the same style. However, we should keep in mind our strong caution regarding the use of AI-generated text. The output should be considered an example and rewritten in one’s own words.

Use role-playing. A popular prompting technique is to ask the model to “act as.” For example: “You are a statistician. Explain p-values in the style of a friendly tutor.” This sets a persona and can affect tone and content (making it more technical or accessible, depending on the role). This is useful for either leading the model to take on a certain voice or enforcing constraints. Another example: “You are an impartial research assistant that only uses evidence-based information. Answer the question …” Such role assignment can sometimes also bypass unhelpful general responses and obtain a more precise answer style.

Find missing pieces. Another useful way to expand our work is to ask AI what we have forgotten. For example, a user could upload their working paper on a given topic and prompt the Gen AI to tell it what critical arguments are missing from the paper. On a simpler level, a user could prompt, “I am going to a conference in London, I packed my laptop, and changes of clothes. What am I missing?”

### 4.4 Interactive iteration

Prompting is not a single request. It is a dialog. If you do not obtain the desired results, refine your question. All Gen AI we are aware of operate inside of ‘chats.’ These are conversation-specific channels. Everything the AI responds to in each of these chats is based on the chat history. Normally, these chats are found by default on the left panel of a Gen AI user interface. Whether Gen AI saves the features of the chat or simply looks at the previous prompts in a conversation matters for the outcomes, and you might have to prompt it to read previous prompts within chats, especially if the chat history is very long or you have waited a long time since last being active in that chat.

Chat channels significantly empower users. This means that you can set up what and how (see 4.1 and 4.2), and this is stored so that you do not need to keep asking. You could ask for a 200-word abstract in Modern Language Association (MLA) Author-Date format, and then a follow up would simply be a refinement, like “please remove sentence X, and add information about the case numbers” and you should again get a (roughly) 200-word abstract in MLA style by default from then on in that chat without asking each time.

Simultaneously, it is important to start new chats for new prompt topics. If you suddenly switch to asking about cats in a chat history about how a correlation works, you are likely to get a response about correlations rather than cats; or some odd mix of the two.

Prompting is iterative within chats, but can also be interactive across platforms. This is essentially what AI Agents like ‘Deep Research’ attempt to do with themselves (several ANNs running simultaneously), but you should also act as an agent seeking to get what you want (from several Gen AIs at once). For example, you might prompt several different Gen AIs (ChatGPT, DeepSeek, and Gemini) simultaneously and extract the most useful results. An agent creates a task list, attempts to execute it, and checks the results against various steps in the process, we find it helpful to keep in mind that both you and the Gen AI can and should do this for optimal results. When you do this it is known as “chain-of-thought” prompting, which calls on the Gen AI to do specific tasks in order that follow a logic or reasoning steps that mirror cognitive tasks ^49^.

To solidify these ideas, let us walk through an example of prompt evolution, as if we are “prompt engineers” ^m^ to refine our query:

Scenario: We want AI to help brainstorm research ideas on how generative AI can be used in adult education. We gave the naive prompt, “Give me research ideas for using AI in adult education.” AI likely gives a few generic ideas, likely surface-level, about AI tutors for adult learners or AI for personalized learning.

We think about how to improve the prompt: “I am looking for research project ideas on applications of generative AI in adult education. Please provide three detailed ideas, each a few sentences long, explaining the idea and why it is significant. Focus on practical use cases in adult learning settings (such as workplace training or continuing education), and make sure the ideas are distinct from each other.” Although this sounds like multiple tasks, they all relate to the core task of listing the three ideas. They fine-tune the task rather than give new tasks. This included several shots. AI should now produce a structured list of the three ideas, each with some details and explanations.

It is possible that these ideas are still somewhat generic. We could add: “For each idea, mention any challenges or research questions that would need to be addressed (e.g., privacy, effectiveness, user engagement).” Here, we take advantage of the fact that chat history is stored. Therefore, we did not need to rewrite the second prompt. AI should now append or integrate challenges into each of the already listed ideas.

Now that we are satisfied with the ideas, we can ask it to produce a report with valid references. This is only possible with a literature database or an Internet search tool. We then prompt: “Name any existing studies or examples that relate to these ideas. Use an Internet search to ensure true, high-quality citations.” We can refine this request until satisfied and then ask for the final report: “Now combine the preceding into a final report with the following components: 1. A creative title, 2. An image on the cover page that captures advances in adult learning, and 3. One page devoted to each of the three project ideas with at least two related citations integrated per project (in author date format plus a link), 4. A paragraph summary at the end about how the three projects all would contribute to improving scientific knowledge in adult education research”. The results are presented in Online Appendix, Prompt 2 ^n^.

This iterative process shows how we can coax AI towards what we need. In each step, we provided more structures or asked for more depth. Finally, we have a finished product that appears as something we can submit as part of our work. However, in this example we would not do that, and instead re-write the entire report, or write one from scratch based on the ideas, and then double check all logic and citations, as this is the best practice and allows us to have our own copyrightable work.

Moreover, looking at the results carefully in Appendix Prompt 2, there is more to do – *more to prompt* in other words. The image was taken from another source, and there may be issues with its use. When clicking on the source, the link does not lead to a website containing the image; therefore, it is unclear whether ChatGPT invented the image. The authors’ date citation request was ignored, perhaps because we also asked to provide links. Refining these steps would require more iterative prompts. We stopped here to show both the pros and cons of Gen AI usage. In reality, as experienced users, we would keep going and refining to get even higher quality outputs. We could also ask for it in the PDF format once we have something better. We have intentionally left this prompting dialog unfinished, to keep users fully aware that without careful checking there are likely mistakes, omissions or errors in Gen AI output.

Prompting is a skill that develops through experimentation. Often, reading what others have done. There are many prompting videos and resources available online. A prompt that works in one context might not directly transfer to another. In teaching or working with colleagues, it can be useful to show how much a prompt can change output quality. For example, giving a poorly specified task versus a well-crafted prompt and comparing the results often convinces people of why prompting matters. In prompting you not just to ask a question, you are guiding a process.

## 5. Conclusion

Artificial intelligence transforms how researchers find information, analyze data, and generate content. For researchers, teachers, and academic staff, the promise of AI in speeding up literature reviews, developing course materials, translation, offering writing assistance, and uncovering patterns in data is fantastic. At the same time, there are pitfalls and great uncertainties regarding when and how to use AI. We attempted to combine our knowledge as regular users of Gen-AI to help researchers stay informed and improve their work.

A crucial lesson is that AI is a tool and not an oracle. AI’s knowledge is based on training data and pattern recognition, not on true comprehension or authority. It can err confidently. One should *trust but verify.* This follows the ideal scientific practice recommended long before AI from a Mertonian-norm perspective ^50^. “Organized skepticism” should be a central motivation for AI outputs. Knowing the basics of how artificial neural networks trained on massive amounts of data (i.e., “LLMs”) helps bolster this skepticism and improves AI outputs and concomitant knowledge production.

The fact that AI is not an oracle, but can behave like one, is dangerous. We may become confused and think that AI is telling us the truth when instead, it is maximizing our desires as extracted from our prompts. This introduces the threat of scientific AI hacking. A researcher might keep prompting until they get the answer they want, in order to achieve a goal like a publication, rather than one that is useful and reliable. AI can also be used to p-hack directly, dredging through data to find unique but not generalizable associations that a researcher can then pretend to have ‘discovered’ in a well-designed hypothesis test. Demystifying why AI might give an incorrect answer, be biased, or even used intentionally to hack should be a core task of learning for academics today.

Understanding the limitations of AI, such as a lack of up-to-date knowledge or inability to reason like a human, empowers users to avoid misusing AI. It is good to offload cognitive burdens, but this should be done in a manner where the human user retains control over every step in the workflow: That they understand (check, vet, interrogate) everything that is being generated. For example, if you do not know how to use R statistical software, then having AI write R code for your research might be a bad idea because you cannot vet it. We believe that it is the task of researchers to focus on how AI can enhance their work, while recognizing that their work will be best when they also understand the underlying mechanisms (methods, theories, literature) of their craft. AI extends and augments what a researcher can do; it is not a replacement for researchers.

Recent and rapidly changing trends shape how researchers use AI tools. Academic publishers and databases have also incorporate AI. For example, Springer experimented with GPT-based assistants on their platforms to help find content. Zotero, an open-source, non-profit reference manager, now has a plugin (“ZoteroGPT”) that can summarize PDFs in your library. We generated a podcast from this study using NotebookLM, which is highly accurate ^o^. Because of these technologies, Gen AI will revolutionize science communication. It can summarize the mountains of research in a way that general audiences of even school children will understand, but the risk of hallucination and misinformation is ever present, and the rate of adoption is so high with Gen AI that there will be no way to vet all these communications ^51^. For example, Robert F. Kennedy Jr. produced a report on children’s health generated from Gen AI with errors and fake citations. ^52^

Science policy organizations encourage the exploration of AI in research. In Germany, initiatives within the National Research Data Infrastructure (NFDI) and in the U.S. within the National Institute of Standards and Technology (NIST) have investigated how AI can help manage and analyze research data. Data management plans are beginning to include sections on AI usage. This means that support for researchers may improve, for example, institutional access to better AI tools that are privacy-compliant, such as Microsoft’s Copilot (provided your institution trusts Microsoft).

New AI-driven platforms for scholarly collaboration are emerging as tools that automatically write the first draft of a literature review section based on a shared library of papers that a team can then edit. These blur the line between source finding and writing. While promising for productivity, they again require that researchers do not skip the understanding part: an AI-generated literature review is only as good as the sources it was given, and cannot interpret significance or context the way a human researcher can.

AI has become an indispensable assistant in the handling of information. By learning what different tools can be offered, researchers can significantly enhance their efficiency. However, the effective use of these tools involves knowing their scope and limits. As the saying goes among statistical modelers: “garbage in, garbage out”. This applies to the quality of the AI augmented research process. It depends on the quality of questions, data sources, training data, and, ultimately, your own judgment. Again, we remind the reader that they are responsible for everything on which they put their names. We cannot stress enough that copying and pasting AI written work into our scholarly works is a dangerous idea and should be avoided at all costs. When using AI-generated text, read every word, and (re-)write your own words to describe what you want.

## Ethical considerations

Ethics and consent were not required.

## Data Availability

No data are associated with this article.

## Footnotes

a
It is perhaps not entirely a coincidence “GPT” is in the name “ChatGPT”. Although this technically refers to “Generative Pre-trained Transformer” it cleverly foreshadows Gen AI’s role in human existence.

b
Online Appendix: https://osf.io/xf52m/ Glossary: https://osf.io/pkec5

c
See for example: https://en.wikipedia.org/wiki/Neural_network_(machine_learning)

d
The number of layers, learning rate, optimization and regularization which are known as *hyperparameters* and are set prior to training, unlike parameters which are adjusted and updated during training.

e
Computer scientists experimented with hybrid models as Gen AI but quickly discovered that the decoder portion was not necessary. It consumed time and resources without improving the results.

f
A German idiom meaning “egg-laying woolly milk sow,” a mythical creature that provides eggs, wool, milk, and meat – in other words, a single source of all needs.

g
Deutsche Forschungsgemeinschaft in German.

h

https://docs.unsloth.ai/get-started/fine-tuning-guide

i
The word “nearly” is crucial here. There are now infamous examples of Gen AI getting specific mathematical calculations wrong. Even after running Python code to get the correct answer, but still insisting their wrong answer is correct. See for example https://i.redd.it/ii5bfbplvf2f1.jpeg. However, look at our example asking to generate the formulas that build a slopes-as-outcomes regression model, they are flawless https://chatgpt.com/share/e/684af1ce-c398-8004-b2b3-6816601648c7.

j

https://osf.io/fguk6

k
Note that output follow proprietary conventions. This means that Gemini will not generate a Word document and instead offers a Google Doc.

l
Use of these words could also increase the usage of computational resources ^48^; however, there are reasons to remain kind and polite in Human-AI interaction because it might prevent degradation of Human-Human kindness in society.

m
We are not particular fans of the term “prompt engineer” as it seems to be a buzzword. We do not see prompting as a skill that requires special job titles. Analogous would be “internet search engineer” which at this point in time sounds absurd as a job title.

n

https://osf.io/fguk6

o

https://notebooklm.google.com/notebook/42571778-12bb-41a1-b6a6-0d52c114d301/audio

p

https://osf.io/fguk6

q
Grok is also a popular Gen AI developed by xAI, an American company. Launched in November 2023, Grok is integrated with the social media platform X (formerly Twitter). It can be tailored to specifications, but is designed initially to be wittier, mimicking social media behavior. This fact and the actions of the company’s founder working against science and science funding are reasons to be skeptical of this product as an academic tool.
