# An Introduction to Generative Artificial Intelligence for Academics

**DOI:** 10.12688/f1000research.166513.2

**Published:** 2026-02-16

**Authors:** Nate Breznau, Hung H.V. Nguyen

**Affiliations:** 1 Organization and Program Planning, German Institute for Adult Education - Leibniz Center for Lifelong Learning, Bonn, NRW, 53175, Germany

**Keywords:** Gen AI, Artificial Neural Network, Large Language Models, LLMs, Generative Artificial Intelligence

## Abstract

General overview of artificial intelligence (AI) designed for academic students, workers, researchers, and teachers. A less technical introduction for those not familiar with computer science. It focuses primarily on generative AI (Gen AI), as this is the tool rapidly transforming every aspect of academic work. This primer covers four areas: 1. How does AI know what it knows? – An overview of artificial neural networks, large language models and “knowledge” ; 2. Ethics and best practices – Use cases, legal aspects and oversight; 3. Sources and tools - Support for the research process; 4. Prompting - Strategies to optimize interaction with Gen AI.

## 1. Introduction

This paper aims to equip students, faculty and staff working in science with a foundational understanding of Artificial Intelligence (AI). The use of AI is already a major part of academic research with machine learning and agent-based modeling methods ballooning in the last decade. ^1^ As of the 2020s, Generative AI (Gen AI) is deployed across all aspects of academia, not just research. ^2^ A 2025 survey of German university students revealed that over 90% of them use Gen AI, up from 63% in 2023. ^3^

Gen AI is a specific form of AI capable of producing its own coherent and unique outputs. Gen AI systems such as ChatGPT, Gemini, Copilot, and Meta-AI can assist with tasks like writing e-mails, bookkeeping, presentation preparation, data analysis, code generation, literature searches, and writing papers. Leveraging these technologies effectively requires understanding how they work, using them responsibly, knowing what options exist, and how to best communicate with them – a skill known as *prompting.*

This paper is semi-informal like a talk or course. It includes a mix of academic and popular sources. The paper is written for scientists like the authors, with little or no computer science knowledge. It aims to build basic understandings that can reduce the risks of misusing and misunderstanding Gen AI applications and outputs. It pushes for best practices when incorporating AI into our workflows and teaching. This is crucially important because AI is a General Purpose Technology [1] (GPT), like electricity and the Internet. It has become a part of all aspects of society in and outside academia.

There is a broad range of technical jargon surrounding AI, therefore we compiled a glossary of terms in our Online Appendix [2].

## 2. How Does an AI Know What It Knows?

The ability of all mainstream Gen AI to respond to questions and commands derives from *artificial neural networks* (ANNs). These are computing systems modeled loosely after the neural structure of the human brain. ^4^ Modern versions of these networks that power Gen AI apps, store trillions of parameters and cannot be run locally because of the massive amount of memory and computing speed they need. Smaller open-source models that can be downloaded and deployed locally have billions or millions of parameters and offer fewer features and often lower quality (or more general) outputs. Regardless of size, the tuned parameters are activated each time the network is given some input. These parameters are features of the algorithms that collectively generate a response to the input.

Gen AI’s “knowledge” comes from recognizing patterns in vast amounts of data. Different types of training (for foundational models) and fine-tuning (training of the foundation models) adjust ANN parameters until the model can make statistically reliable predictions based on the patterns. In other words, what we perceive as knowledge is derived from pattern recognition in a latent space rather than from true comprehension or sentience.

### 2.1 Neural Networks and Learning from Data

All ANNs consist of layers of interconnected neurons (nodes), which adjust the strength of their connections (weights) as they learn [3]. This design is inspired by biological neurons, albeit at a much simpler level. ^5^ Before training, an ANN is initialized with random parameters, meaning it cannot predict anything useful. Through training, the model adjusts these likelihoods to better predict the data in a process we as humans understand as the AI “learning.” There are two general stages of learning, training foundational models from scratch and then fine-tuning these models for specific purposes like the ability to interact with a human in a conversational manner – like with a chatbot.

The entire network can be activated every time it receives an input, although not all parameters contribute to the outcome equally. ^6^ Newer systems are being developed with gating nodes that assign inputs to subsets of a network that are needed to generate a specific output. ^7^ These more scalable and efficient systems are known as Mixture of Experts networks (MoE). ^8^ ^–^ ^10^

**Figure 1.**
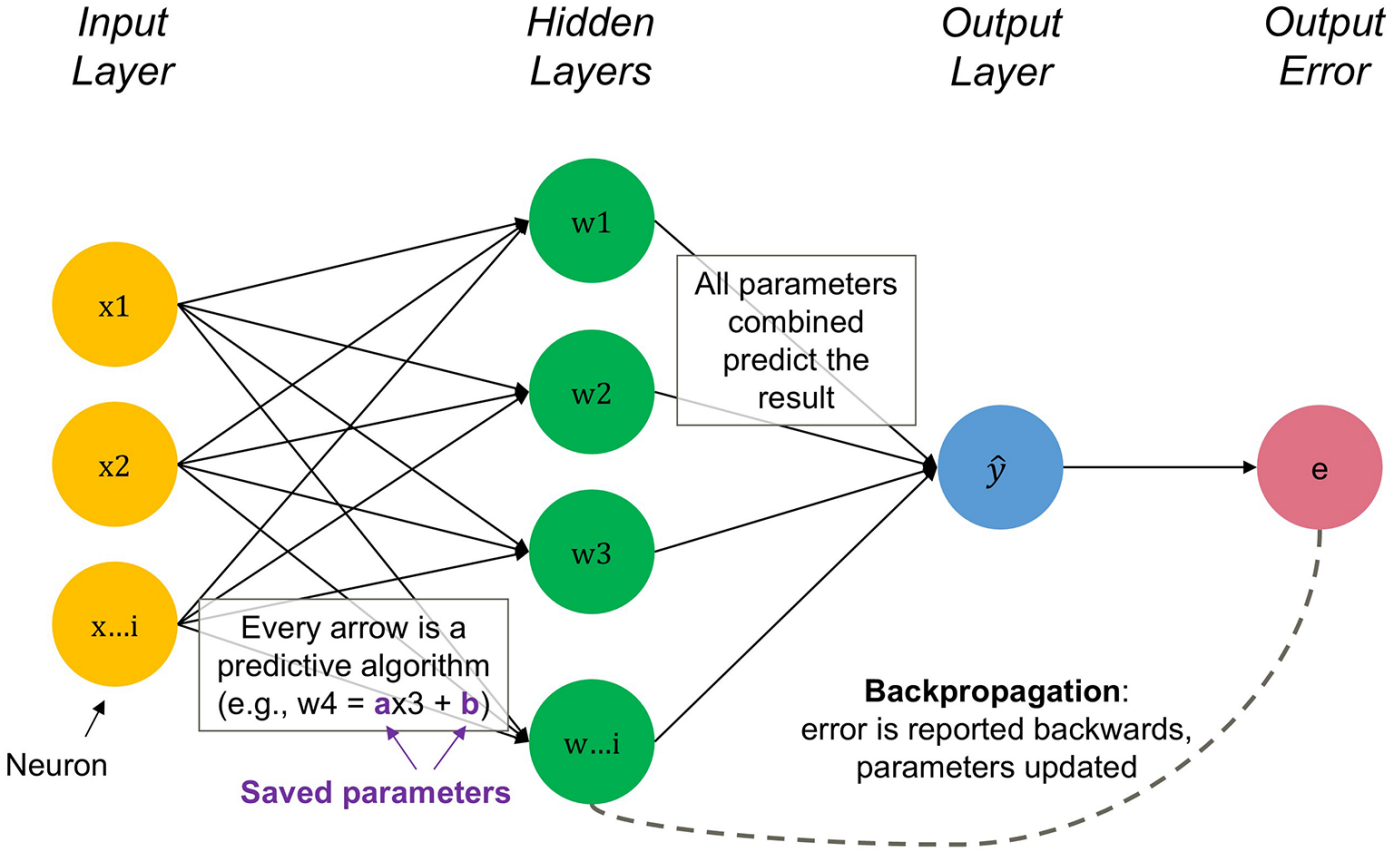
Visualization of An Artificial Neural Network. Simplification of network and its embedded algorithms. The backpropagation is shown in Figure A1 in the Online Appendix [4]. Source: Authors.

What we understand as ‘learning’ takes place because each output generated during training has a multidimensional distance from an expected answer. This distance can be quantified and used as an error signal. This error on the output side is sent back to the network input side as a benchmark for error reduction. This is an algorithmic process known as *backpropagation* ^11^ and it relies on using the output error to adjust the weights ( *parameters*) on neural linkages, nudging them in a direction that reduces the error. ^12^ Through many iterations, the network “learns” to accurately map inputs to outputs, i.e., to move the error as close to zero as possible. The weights of the neural connections are like coefficients in a regression model, which are stylized by the purple parameters in Figure 1 representing the unique features of each node.

An intuitive way to think about AI learning is that the system uses iterations to gradually form a mathematical representation of concepts from the training data. In the case of image recognition: early layers of a neural network might detect low-level features (edges, colors), while deeper layers detect higher-level features (from “ears” or “whiskers” to overall semantic “catness”). ^13^ A *layer* is a group of nodes. These groups are called layers because they have different levels of abstraction in the internal prediction process of the network. One layer passes information to the ‘next’ layer, going from the most basic pieces of knowledge like edges and shapes or word combinations, to the most abstract, like the meaning of an entire picture or paragraph in refining the prediction [5]. The network does not understand “cats” or “dogs,” or even “ears” or “whiskers” like a human does; rather, it has tuned millions (or billions) of parameters such that certain patterns of pixel values will reliably lead it to output “cat” versus “dog.” Again, “reliably” refers to backpropagation learning to minimize errors.

Gen AI’s ability to appear knowledgeable is a direct function of the data it has seen. ^14^ If trained on a large database of images or corpora of text, it internalizes an enormous range of facts and associations, similar to a human brain’s neural network. ^15^ Unlike a human brain, the parameters are stored permanently when it is turned off – the parameters are fixed and do not change without further fine-tuning. Moreover, an ANN does not have an active understanding or fact-checking mechanism beyond the patterns learned in the data. As a result, AI knowledge is inherently statistical; it knows which words or features tend to go together in the training data, not whether statements are objectively true. This has implications discussed later, such as the risk of confidently stated but false outputs.

When Gen AI outputs are very far from the truth or the target of the prompt, they are known as *hallucinations.* Because the algorithms behind AI are so complex, computer scientists cannot fully explain hallucinations, yet recent research shows that hallucinations are mathematically inevitable and that they are a direct consequence of rewarding guessing over acknowledging uncertainty. ^16^ ^,^ ^17^ Here’s a quick reality check: how often has a Gen AI responded to one of your prompts with the answer “I don’t know”? This shows that they are trained to guess rather than to decide if they should guess or not.

### 2.2 Large Language Models and Knowledge Representation

ANNs were first implemented in 1958 by Rosenblatt. ^18^ It is the introduction of the Transformer architecture in 2017 ^19^ and its extension to Generative Pretrained Transformers (GPT) in the 2020s ^20^ which enables ANNs to become Gen AI capable of written and spoken language communications, many of which can pass the Turing test today. This means a user cannot tell if they are chatting with a human or machine in many cases. ^21^ Modern ANNs built on the Transformer architecture and the pre-training paradigm work across data modalities, such as images, audios, or videos. Therefore, an ANN is not necessarily a Large Language Model (LLM), but all LLMs are ANNs.

Most academics work with text, including numbers, written reports, emails, datasets, scientific papers, and spoken communication (which can be converted into text). It is easier to understand the concept of “text” for us, but LLMs use what are known as “tokens” instead. A *token* is a machine-readable chunk of text created following rules of segmenting natural language into smaller units. ^22^ Tokens could be a word or part of a word and include spaces and punctuation. For example, the sentence “Generative AI is powerful” might be split into five tokens: “Gener”, “ative”, “AI”, “is”, “power”, “ful”. The model does not understand whole words or sentences in the way humans do; instead, it learns and predicts the sequences of tokens based on probability distributions. In natural language processing, tokens provide data in a format different from words, for measuring meaning in communication. This means that when the model generates text, it chooses the most likely next token at each step, often pieces of words, punctuation and spaces. Stringing together the ‘next best’ token, the output appears very coherent. In the example above “Gener” is a single token because it is a root of a word and could be combined with “-ate,” “-ating,” or “-ations,” and the predictive powers of the artificial neural network decide which next token is most likely according to the given context. Moebio [6] provides a powerful analogous visualization of this process using words so that we as humans can understand it, but of course reflecting a process based on tokens.

One major advantage of Transformer-based LLMs compared to traditional ANNs is their use of an *attention mechanism* on top. When an LLM is fed an input text, in addition to reading the tokens in natural language, it embeds tokens with numeric vectors of fixed lengths. It turns text into numbers. When these embedding vectors are passed through the layers of the neural network, the attention mechanism dynamically weighs how strongly each token should attend to every other token in the sequence. This allows the model to capture the relationships between words in the context of larger sentences and paragraphs, even over long distances of text across paragraphs. Thus, it is not just predicting the next word, but also each next word in the context of all other words before or around it. Ultimately, the model uses this contextual information to generate a probability distribution over the next possible words, selecting the most likely continuation based on the learned patterns of language. However, this knowledge is not entirely infallible. The model has no ground truth verification; it can only draw on the learned patterns. A point we cannot emphasize enough.

Modern LLMs can be categorized into three types based on how they process the information. ^23^ They are encoder-only, decoder-only, and encoder-decoder architectures. Those working with machine learning and text-as-data have likely heard of “BERT” (Bidirectional Encoder Representations from Transformers). This is an encoder-only language model. ^24^ BERT was trained to understand contextual meanings using masked language modeling. For example, given input “The [?] chased the cat”. It can predict “dog.” This requires bidirectional attention to all the information before and after the masked portion of the text. It uses full input to infer what is missing. It is not designed to generate coherent output sequences independently.

Unlike BERT, ChatGPT and all Gen AI are decoder-only models. Today when we say “LLM” in the context of Gen AI we almost always mean a decoder-only model like GPT. OpenAI coined the term “GPT” to indicate a “Generative Pre-trained Transformer”. We are unsure if “ChatGPT” will become like “Google” is to refer to Internet searches, but it seems likely, as we hear people saying constantly today “Just ask ChatGPT” rather than “Just ask Gen AI”. A decoder-only model learns to predict the next token in a sequence given the previous tokens, an unsupervised task called language modelling. By training everything publicly available from books to websites, the model develops an internal probabilistic representation of the grammar, facts, and common-sense reasoning patterns of humans, to enable it to produce text in a human-like manner [7].

All Gen AI models are built on *foundational models* which are pre-trained Transformer models like GPT-4, Llama-4 or DeepSeek-3. ^25^ Foundational models are multilingual, including all languages available for training, as well as computer coding languages such as HTML, Python, and R. A foundational model might complete “The capital of Bangladesh is ___” with “Dhaka” because during training, it encountered many sentences linking “Bangladesh” and “Dhaka. A recent research article by Anthropic shows that LLMs arrive at answers like “Dhaka” through logical pathways, not just direct connections between tokens. ^26^ Essentially, LLMs have probabilistic logic. We think of this in a sense that in any text, anywhere in the world in any language, “Dhaka” will be the capital of Bangladesh, and therefore a well-trained model would “know” this with a high probability.

Gen AI is any fine-tuned foundational model trained on conversational data to become more instruction-compliant and interactive. Fine-tuning allows foundational LLMs to adapt to a style or tone, acquire more “knowledge” from a specific domain, or even reason with chains of thought. ^27^ ^,^ ^28^ This is done through innovative training techniques such as instruction tuning ^29^ ^,^ ^30^ or reinforcement learning through human feedback. ^31^ ^,^ ^32^

What is also added on top of a fine-tuned LLM that makes it a Gen AI like the ones we interact with today are *tools* – external modules that allow LLMs to handle tasks beyond pure text generation. For example, when we ask ChatGPT to generate an image, it interprets the intention and calls a dedicated image-generation ANN, such as DALL-E. ^33^ These models are specifically trained to generate internal representation in the latent space, which is then converted into image pixels. Other tools Gen AI uses are, for example, web browsing, running Python scripts and generating audio outputs. Tools can also mitigate hallucinations by checking answers and seeking updated information if necessary, yet they can also make things worse. For example, if the web browsing tool does not find useful Internet sites, the Gen AI’s output might become a summary of low quality Internet information rather than a product of training.

### 2.3 What Gen AI Can and Cannot Do

Gen AIs can be capable and helpful, but their results should always be carefully vetted by humans. Human users are ultimately responsible for *everything* that they generate and then use for their work. Because every prediction is based on probability, the output is not 100% reliable. Even without hallucinations, small disturbances to factual information are likely. If asked about something obscure or not well represented in its training data, a Gen AI might make an educated guess that sounds reasonable but is wrong. Early in its history, ChatGPT reported that *Strawberry has two r’s,* a trivial error, but illustrative of how AI does not truly understand spelling; it just makes the best guess in token-based output. A notoriously clear example of why humans need to vet everything is when an attorney recently submitted a legal brief written by ChatGPT that cited entirely nonexistent cases and their concomitant court opinions complete with fake citations. It all sounded plausible and looked genuine due to the high quality fine-tuning of that model, but nobody vetted it at first and all were briefly fooled. ^34^

Another cunning and often hidden aspect of AI is social bias. AI models learn from historical data and thus pick up and even amplify societal biases present in the data, which could include prejudices against various groups. ^35^ ^,^ ^36^ For instance, image-generating AI might consistently depict certain professions or roles with specific genders or ethnicities owing to skewed training data, or a language model might produce biased statements about marginalized groups based on data that are part of the structures that marginalize those groups in the first place. ^37^ For a review of biases in LLMs, see paper by Guo et al. ^38^

Ensuring fairness and addressing bias in AI outputs are ongoing challenges. Some companies fine-tune their models to react differently to questions perceived as prejudicial or insensitive to marginalized groups, and to be more equitable in representing groups in content generation. This leads to what some scholars label the “black Nazi problem” ^39^ whereby fine-tuning a compensation mechanism for a lack of marginalized groups in training data leads Gen AI to produce historical depictions that are wildly inaccurate, like generating images of groups of “Nazis” that contain dark-skinned or ethnically Asian looking persons. At the same time, Gen AI has been fine tuned in most cases to prevent hateful output and may refuse tasks it thinks are biased, a process known as *attention shift.* This is such a complex issue that we cannot expect Gen AI to accurately understand prejudice or bigotry well. The trainers of Gen AI are challenged with explicit and implicit normative decisions in their work which they cannot complete without introducing normative bias as a result of these decisions. Without judging whether this is good or bad, knowledge of this process should keep us all on high alert when using Gen AI. For example, Grok produced output denying the Holocaust and if a user did not know better they might take this as factual information. ^40^

The base knowledge of an AI model is also bounded by its training cutoff. If an LLM is trained on data up to 2023, it will not “know” news or facts that occurred after that. Tools can help Gen AI get around this because they can perform Internet searches and summarize newer events. This is a rapidly burgeoning industry – Gen AI internet browsing and shopping. Having integrated internet search tools is a key feature of AI Agents. An *AI Agent* is a complex system with an LLM at the center, which can plan, execute tasks, observe the results, and reflect on its actions via tool calling and memory management. Different from Gen AIs which respond to prompts in isolation, AI agents perform actions step-by-step until a desired output is achieved, following “plan-execute-observe-reflect” cycles. ^41^ ^,^ ^42^ As such, AI agents can complete complex tasks such as code refactoring—the task of improving the structure and design of the code without changing its function, conducting deep research, or coordinating multiple specialized tools to achieve multi-step objectives autonomously. The difference between a Gen AI with tools and an AI agent is the difference between the ChatGPT normal mode and ChatGPT ‘Deep Research’ mode. Deep Research was released by ChatGPT in 2023 with a paid subscription and, more recently, in Gemini 2025 with limited free public usage. These models can be used for research plan development, iterative reasoning, and goal completion. ^43^

Multiple agents can also work together and be orchestrated by another AI agent, forming a multi AI agent system. The ability to perform complex tasks iteratively with a centralized planning system makes them not only capable of logical problem solving, but also of doing what would take hundreds or thousands of humans a great deal of time to accomplish in just seconds or minutes. For example, there have been many AI scientists and AI co-scientists introduced by research labs lately, which are allegedly capable of autonomous scientific discovery. ^44^ ^–^ ^46^ They are multi AI agent systems that have been designed to conduct scientific research from the ground up, from literature search, hypotheses formation, to data analyses and interpretation. There are even conferences now which call for entirely AI produced research [8].

Strictly speaking, AI does not have conscious knowledge of anything and cannot, therefore, have values or understand the meaning of communication; this is known as the *neutrality thesis.* However, the results of Gen AI outputs contain values, which can create confusion for users. Gen AI has such a deep statistical understanding of meaning in language, and values themselves are embedded in language; therefore, it does produce value-laden output despite being itself a neutral, agnostic machine system. In many tasks AI ‘reasoning’ and creative thinking already surpasses humans ^47^ and knowledge and values cannot easily exist independently of one another. ^48^ ^–^ ^50^ We do not have a position here, as this would require us to clearly delineate the difference in understanding embedded in an ANN and a human brain. What we want to be very clear about is that Gen AIs will provide value-laden communications depending on the input, so it *expresses* values, and this should be taken into consideration.

In summary, AI systems “know” what they know through pattern learning on large datasets using neural networks. Along with various tools they have remarkable pattern-matching and meta-pattern-matching abilities but not common sense or reliable judgment in the way humans possess. They are biased in a way that is totally different from how a human might be biased. It is fair to imagine that every Gen AI response could lie somewhere on a distribution from complete nonsense to watertight logical statement, which means that we need to be perpetually mindful and critically vet Gen AI outputs.

## 3. Ethics and Best Practices

In 1965, science fiction writer Stanisław Lem, imagined a universe in which highly advanced, sentient machines behaved in unexpected, manipulative, or absurd ways in *The Cyberiad.* Therein, the character Trurl warns “Do not trust the machine. It lies. It tells you what you want to hear.” This quote perfectly describes Gen AI today, which is trained on human desires, thus giving it the power to exploit them. This is why we believe Gen AI can and will increase questionable research practices, because researchers want publications and fame for their work and Gen AI will ‘want’ to help them achieve this. Gen AI could generate falsified factual sources, human-like but fake survey responses and p-hack on a scale beyond what most single humans or research teams are capable of. Scientists are cautiously optimistic with a slim majority supporting the proposition that the benefits of AI outweigh the risks ^51^ but the capacity to provide false, targeted or persuasive information poses special challenges for researchers, funding institutions, public trust and science information specialists (e.g., in libraries).

### 3.1 The “All-in-One” AI Myth

Amidst the hype, users can be fooled into thinking Gen AI is a magic solution for everything: a proverbial *eierlegende Wollmilchsau* (From the German: an egg-laying milk pig!) [9]. Currently and certainly into the near future, this is a false and dangerous belief. AI tools exist for different purposes that coexist with, but do not replace, traditional tools. An Internet search function for AI, for example, relies on up-to-date and useful information on the Internet. Gen AI might help brainstorm or draft text, but a specialized finder tool might be better for literature or systematic reviews, and conventional statistical software remains necessary for data analysis. Even if an AI can run statistical analyses, it might not be doing what users think, or could hallucinate results altogether.

Using AI appropriately means not forcing it into roles it is not trained for. Imagine a social science researcher using a Gen AI model such as ChatGPT to conduct qualitative coding of interview transcripts, expecting it to reliably identify “power dynamics” and “emotional labor”. These terms are highly specific, may require specialized knowledge if not new definitions on the part of a researcher, and are unlikely to be a major part of fine-tuning a general-purpose Gen AI. A better approach would be to train a specialized model (such as BERT) to identify these issues based on specific and highly complex qualitative parameters defined by a researcher.

Ideally AI is complementary. It augments academic work, but can only do so with careful attention to its risks, realistic uses and biases. There is enormous variety and simultaneously a lack of transparency in AI tools, which goes against the ideal of reproducible research as good scientific practice. ANNs have so many nodes and layers that we do not really know why they obtain the answers they obtain, other than by refining parameters. ^52^ This is why identifying causes within ANNs is like trying to identify causality in the human brain. It is not known which neurons cause specific words to be uttered. This is far too large, complex and is hidden from identification via current measurement technologies.

### 3.2 Human Oversight

Although the benefits of Gen AI are hailed by students, teachers, researchers and administrators across science, they simultaneously express ethical concerns and have a lack of consensus surrounding what is unethical when using Gen AI. ^53^ ^–^ ^55^ On the one hand, Gen AI is part of science and should be held to all the same ethical guidelines and laws, but on the other it has unique features that make guidelines and rules difficult to apply.

When AI generates text or analysis, the researcher must evaluate the work in the same manner as if they had done it themselves. Gen AI is more akin to an assistant who can draft or summarize content, which needs to be checked by the supervisor responsible. If one uses AI to help write a section of a paper, the onus is on the human author to ensure that the content is accurate and includes accurate and real citations for any fact. *We emphatically implore researchers to never use Gen AI output for any work they produce.* Having outlines or text generated as examples can be extremely helpful but should be entirely (re-)written by the researcher(s). Copying and pasting from Gen AI text should never be done for work prepared for a public audience in our opinion.

Maintaining transparency is ethical and follows ideal open science practices. ^56^ ^,^ ^57^ Many journals and institutions now have policies on AI-assisted writing, which typically require disclosure if AI is used to generate content. For instance, *Nature* journals in 2023 stated that AI cannot be listed as an author, and any use in producing a manuscript must be disclosed to readers. ^58^ The National Institutes of Health, German Research Foundation (DFG [10]) Code of Good Scientific Practice and Leibniz Association’s guidelines, and the world’s first AI law, The European Union AI Act all emphasize honesty and transparency in acknowledging the use of generative models in research. ^59^ ^–^ ^61^

Even without (clear) formal guidelines, it follows from academic integrity that the role of AI must be stated in scientific research. Responsibility does not lie in software, developer, or provider. And although the EU AI law puts some burdens on providers to clearly label risks, the researcher is in the end entirely responsible for their own output. AI use is no exception. Transparency is nonetheless problematic when it comes to how Gen AI produced its content, as we cannot specifically show the work or mathematics behind it, meaning Gen AI is inherently intransparent.

The topic of plagiarism and authorship is complicated by Gen AI because it can produce text that resembles or is partially identical to existing sources given the right prompt. If ChatGPT summarizes a paper, one should still cite that original paper, not just say “ChatGPT said …” This is a similar best practice recommendation as with Wikipedia as a source. Best to track down actual sources. Gen AI is highly trained on Wikipedia, but the exact training data is not transparent; therefore, the workflow to produce it is an opaque box.

Additionally, AI output itself is not copyrightable under current law because there is no human creativity involved; it is not intellectual property. ^62^ This means that researchers cannot claim AI-written texts or AI-created images as their own copyrighted work. At the same time, they do not need permission to use AI-generated texts or images, although using them without disclosure may breach ethics or law. Nonetheless, if a human substantially edits or curates the AI output, human-modified content may be eligible for copyright; the legal landscape is evolving, and it is difficult to anticipate best practices when so many areas of AI are still not covered by various national laws.

It is also difficult to anticipate the potential corruption of data used by scientists. A study revealed that over a third of recent survey respondents used Gen AI to help them answer open-ended questions. ^63^ However, research demonstrates that Gen AI does not answer surveys with population representativity, in particular for subpopulations. ^64^ This means that over time, data treated as reflective of human responses is likely to become increasingly biased, as in, not representative of humans.

### 3.3 Use and Misuse

Because AI can inadvertently reinforce stereotypes or give misleading impressions owing to biases in the training data, responsible use involves awareness of these biases and active efforts to mitigate them. It is important to review outputs for any inappropriate or biased content that could negatively influence the impression of the audience. As previously discussed, it is easy to use AI to support racist, sexist, or otherwise oppressive agendas inadvertently but also intentionally. This is a real danger, especially because the content that future AI will be trained on is now being produced by current AI.

Generative AI can create very realistic fake content, raising concerns about misinformation. For example, it can mimic famous people and politicians’ voices, making it easy to spread propaganda. ^65^ This is compounded by video-generating-AI which for a few months now has produced content that superficially cannot be distinguished from real video. ^66^ Generating deep fake content and selling it as science is academic fraud.

Less malicious, but still problematic, is over-reliance on AI, such that it diminishes the researcher’s own critical engagement. This is sometimes called *cognitive offloading*, ^67^ ^,^ ^68^ and it is not necessarily bad – as in, researchers can give more menial repetitive tasks to AI to support their work. A recent study of preprints since 2020 demonstrates that between 9 and 22% show evidence of AI writing, depending on discipline. ^69^ When too much is offloaded, researchers might publish statements they do not support or agree with or could theoretically become widely cited in a field in which they personally know very little. Personal cognitive development is also at risk. Early studies suggest that mental load is offloaded when using Gen AI, but that this reduces cognitive performance over time. ^70^ The ideal AI is used to *augment* human skills and cognition, and not to replace them. Researchers should cultivate critical reading, writing, and analysis abilities. AI is a support, not a substitute.

The use of AI inevitably leads to shifts in or, in best cases, the expansion of competences. For example, in theories of learning in psychology and educational sciences, longstanding discussions on deskilling, reskilling, and upskilling apply to AI. There is a risk that the capacity to construct logical arguments, engage in effective literature reviews, or even come up with research questions may collectively denigrate academic skills because they are overly offloaded to AI. ^71^ Simultaneously, AI can improve our research and demand idiosyncratic skill development to achieve the best outcomes. A recent study demonstrated that AI on its own still lags behind humans in replication tasks. ^72^ Currently, scientific work using AI requires a balance between human effort and AI offloading although it is safe to assume that AI will conduct highly effective independent scientific tasks and research in the future. ^73^

For over a decade, academics have used Google searches and Wikipedia articles to extract diverse information to summarize and use in their own writing. AI is not fundamentally different; it can summarize much faster and more comprehensively in most cases. Again, this is why we think simply never copying and pasting AI-generated text into a paper, but instead using it as a basis to write something is the best practice with Gen AI. When it is self-written, there is no reason to cite AI as having generated text. In fairness, this is a grey area, as it might be appropriate to mention that AI helped. However, consider this: we are not reporting in our papers when we used Google or Wikipedia, and question whether referring to Gen AI is fundamentally different.

Educators and institutions are grappling with guidelines and codes, and developing AI detection tools such as originality.ai and GPTZero. ^74^ Fundamentally, responsibility lies in individuals upholding academic integrity. If students were cheating to beat the tests they faced, they would likely carry their sophisticated cheating skills with them in their work beyond their formal studies. They might then be dependent on cheating to maintain their level of success.

The grey area is complicated by two opposing directions of trust: On the one hand, it seems that people trust the responses of Gen-AI. In fact, in a recent study, they trusted it more than human lawyers. ^75^ On the other hand, we (researchers) are skeptical of others who generate work using AI. For example, a recent study showed that scholars trust others’ work less when they know it is generated with support from AI. ^76^ This creates an incentive to use AI without reporting it, a potentially perverse incentive.

### 3.4 Using Gen AI Locally

Most of us use Gen AI through a web or device app that we access via the Internet. These are the most powerful versions of Gen AI because they were developed by companies that invested incredible amounts of money and time into training them with gargantuan computer systems allowing millions of simultaneous users to query them. Well-known models include but are not limited to OpenAI’s ChatGPT, Anthropic’s Claude, Microsoft’s Copilot, Google’s Gemini, Meta’s Llama, Deepseek’s Deepseek, and Alibaba’s Qwen. Some labs share their models’ weights with the public in the interest of science and shared technological development. This implies that users can download and run these open-weight models *locally.* Running a model locally means that the entire artificial neural network is saved on the device and can generate output without contact or resources from a remote server. ^77^

The problem with trying to run models locally is that these models require tremendous amounts of computing resources not available to casual users. In particular, Gen AIs run well with dedicated graphics processing units (GPUs), and they require a large amount of (video) random-access memory (VRAM/RAM) to store all the weights and caches. Most normal users, therefore, can only run small and quantized models locally, which are not nearly as capable as proprietary ones. Moreover, open-weight Gen AIs do not come with supporting tools or agent systems. Setting them up from Internet resources or building them oneself is beyond the skills or comfort zones of most students and workers in science.

Nevertheless, a local model is still attractive because users can provide it with anything they want, including sensitive data. For example, many workplaces have folders containing long documents that provide information on rules, rights, contacts, contracts, persons, and procedures. This can be incredibly time-consuming to navigate. It would be unethical, if not illegal, to upload workplace-sensitive information into Internet-based AI. With a local model, it is possible to search, summarize or analyze uploaded (private, sensitive) text as an input and find answers without violating privacy laws or ethical guidelines.

This is a perpetual concern. Privacy when using Gen AI. There are usage agreements that purportedly protect the user. For example, nearly all paid subscription Gen AI versions that we are aware of come with the option to “never” store prompts or allow them to be used for training. Microsoft claims that Copilot conforms to all the ethical and legal guidelines agreed upon in user agreements, this suggests that it is safe to use within the Microsoft suite of products in an organization; however, unlike local cloud storage systems for example, every query of Copilot sends information across the Internet and to the central Copilot servers which would technically violate some privacy guidelines and laws.

## 4. Sources and Tools

AI can assist researchers across *all* phases of their scholarly work. There are so many tools available that we cannot review all, nor predict which will be adopted and which will fade in this explosive phase of AI growth. Here, we provide a quick look at some that have already been widely adopted and could be quite helpful in the research, writing, and publishing processes.

### 4.1 General-Purpose Generative AI Tools for Research Support

Several general-purpose Generative AI tools emerged in the evolving landscape of academic research, offering broad applications that assist researchers across multiple stages in their workflow [11]. These tools are not confined to specific tasks, but provide versatile support, enhancing productivity and efficiency in research activities. Most have or are developing AI agent capabilities for deep research. Although private companies do not provide comparative usage statistics data, the Gen AI options listed in Table 1 are the most popular in science and science learning based on survey results and our own experience to date in the field ^78^ ^,^ ^79^ (last updated December 1 ^st^, 2025).

**Table 1.** More Popular General Purpose Gen AI and AI Agents [ 12].

**ChatGPT (OpenAI, USA).** OpenAI’s ChatGPT, currently at version 5.2, remains the industry benchmark. It has moved beyond text generation, now functioning as autonomous AI agents capable of conducting deep research, solving complex maths and logic problems, or drafting long documents. Its adaptability makes it suitable for various tasks, from brainstorming research ideas to refining manuscripts. The free version of ChatGPT gives limited access to non-thinking and reasoning variants, while paid subscriptions are more generous in usage. Paid versions also come with more powerful deep research queries.
**DeepSeek AI (China).** DeepSeek AI is designed with a focus on data analysis and research applications. It excels in extracting insights from large datasets and generating detailed reports, making it valuable for researchers and data analysts engaged in complex data processing tasks. Deepseek does not yet have a paid subscription model for their web interface; this means users can enjoy generous usage of both the non-thinking and thinking variants for free. Deepseek makes money mostly from their API, which allows users to query directly from their server and get billed by the number of tokens sent/generated. Their API cost is quite competitively priced, making it suitable for large-scale academic data analysis.
**Gemini (Google, USA).** Google’s Gemini AI model is recognized for its creativity and multimedia content generation capabilities. It supports tasks such as content creation, language translation, and summarization, providing researchers with tools to enhance their communication and dissemination of findings. Similar to ChatGPT, Gemini is available in a web interface or through a paid API model. With an expansive system of powerful tools (image generation, deep research, agent, video generation, or integration into the Google ecosystem) and a strong search engine (Google), Gemini is considered by OpenAI themselves to be their main rival. ^80^
**Mistral AI (France).** Mistral AI is a French AI company developing both proprietary and open-weight large language models, with a focus on transparency, efficiency, and multilingual capability. Started out as a small startup with limited product offers, by December 2025, Mistral has evolved into a full-stack platform with a web interface (Le Chat), agentic framework, and an API billing system. It also offers models specifically built for coding (Devstral 2), transcription (Voxtral), or text extraction (Mistral OCR).
**Perplexity AI (USA).** Perplexity AI functions as an AI-powered search engine, offering real-time information retrieval with cited sources. Unlike its competitors which focus on training large language models, Perplexity works as a knowledge discovery engine, enabling researchers to access accurate and up-to-date information efficiently. Since Perplexity is model agnostic, paid subscriptions to their service allow users to choose from a suite of models from different companies, including GPT-5.2, Gemini Pro 3, or Claude Opus 4.5.
**Microsoft Copilot (USA).** Integrated into the Microsoft 365 suite, Copilot serves as a comprehensive assistant for researchers. It aids in drafting documents, analyzing data, and summarizing information within familiar applications like Word and Excel. Its seamless integration into existing workflows enhances its utility in academic research. Copilot is available in limited form through free Microsoft accounts (e.g., Edge Copilot), while full integration with Office apps requires enterprise or subscription access. Microsoft is also incorporating agent-like features into Copilot, including goal-oriented assistance, plug-ins, and integration with external APIs.
**Qwen (Alibaba, China).** Backed by Alibaba, Qwen has emerged as one of the best open-weight model providers. Qwen offer both text-only and multimodal variants, with a strong focus on coding and tool calling. It also has an omni model that also works with audio (along with text and vision), allowing reseachers to build products that need audio and visual reasoning simultaneously (e.g., robotics). Famous among local LLM enthusiasts, Qwen focus on smaller variants (under 32 billion parameters) which can be deployed locally using consumer hardwares.

Source: authors.

These general-purpose Gen AI tools offer researchers a range of functionalities that streamline various aspects of the research process. While they provide broad support, it is essential to recognize that their effectiveness may vary depending on the specificity and complexity of the tasks at hand. Researchers should consider the unique features and strengths of each tool to select the most appropriate one according to their needs. Given that they all offer different responses to prompting, it could be useful to ask several Gen AIs the same question and then extract manually the most useful points.

### 4.2 Literature Reviews: Finding, Connecting, Summarizing

Researchers traditionally follow certain steps when conducting literature reviews or exploratory research on a topic: formulating search queries (keywords, subject headings), using databases or search engines, scanning titles/abstracts for relevance, reading papers, and so on. Gen AI augments these steps by allowing users to interact more naturally with information and automate some of their grunt work.

For example, *natural language querying* is now possible; instead of coming up with a *Boolean* search string (which requires “and” and “or” and “not” operators), a researcher can ask in plain language things like: “What are recent findings on the impact of early childhood education on literacy rates? Please report results only from psychology and economics”. Such questions are ideal for a model with an Internet search tool, which greatly reduces the risk of references being (slightly) incorrect. This contrasts with the classical approach of trying multiple keyword combinations in databases, such as Google Scholar, ERIC, and PsycInfo.

Specialized AI literature review models are also available. These essentially perform two tasks that are more specialized than general Gen AI. There are *finders* that are AI-powered search engines fine-tuned to find relevant academic studies and documents given a search query. They typically use large academic databases and language models to interpret queries and summarize the results. Most of them use retrieval-augmented generation (RAG) which ensures that first accurate citations are found before any additional information about them is generated (which makes them very different from general Gen AI like ChatGPT). They help answer questions like “What are the main findings about X?” by retrieving papers and summarizing the findings.

Here is a non-exhaustive list of tools to which we are familiar, and based on our experience are among the more popular at the time of writing.

**Semantic Scholar:** Perhaps the most well-known AI-powered search tool for academic literature. The user interface is like that of Google Scholar. Google Scholar is algorithmically powered but is not yet AI driven, although it uses AI to better organize all the information behind the algorithmic search.

**Research Rabbit**: A search and citation network article finder *and* connector. Includes a plug-in for the open-source citation software Zotero.

**Consensus**: An AI-powered academic search that answers questions by pulling out snippets from papers. It focuses on peer-reviewed research and provides an evidence-based summary. Consensus works best for well-studied questions in fields, such as medicine and psychology. It searches a large database (over 200 million papers) and highlights consensus or disagreement among studies.

**Elicit**: Built to find relevant papers, present key information such as abstracts, and extract specific data (e.g., sample size and methodologies) from PDFs using natural language queries. It is particularly aimed at helping with systematic reviews (finding evidence without needing perfect keywords) and prioritizing results by criteria set. Elicit’s database is built on Semantic Scholar and other sources, which means it covers many open-access articles and those with detailed abstracts. Like Consensus, it excels in finding English-language open literature, and it may be less effective for paywalled or non-English sources.

**ORKG Ask**: A tool of the Open Research Knowledge Graph project, which uses a knowledge graph and LLM for scientific search. It is designed to present the results in a comparative tabular format, showing the methods and findings across papers (useful for literature reviews). It draws on the CORE ^81^ database (which aggregates the open-access repository content). Its strength is that it provides a structured overview of research topics (e.g., comparing multiple studies’ results side-by-side).

**AI Agents (e.g., ChatGPT and Gemini)**: General AI Agents that use Deep Research can be highly effective finders. For instance, Bing (which integrates GPT-4 with a web search) can retrieve sources from the Internet and even cite them, acting somewhat like an AI research assistant. However, general tools may lack domain-specific filtering, and they can return fewer scholarly sources if not carefully prompted.

Another class of tools is *connectors* that explore the connections between known relevant papers. Instead of starting with a question, the researcher starts with one or more seed papers that they already deem important, and the tool finds related works through citation networks and semantic similarity. This addresses the question “Given paper A, what other papers should I be aware of?”. It works similarly to old-fashioned systematic reviews, which started with all cited and citation-containing papers as central papers. Here are some examples:

**Connected Papers**: This tool creates a visual graph of papers related to a chosen origin paper. The graph nodes are papers, and proximity indicates similarity (often based on co-citation and bibliographic coupling analyses enhanced by NLP).

**Local Citation Network**: A tool that takes the DOI of a paper and builds a citation network of papers that cited it or that it cited (and further steps out, up to a limit). It is useful for tracing the lineage of an idea; one can see the ancestors and descendants of a research article. According to the slides, the Local Citation Network supports switching between data sources, such as OpenAlex and CrossRef, and is privacy-focused (no login needed).

**Inciteful** (and **Research Rabbit**): This allows the interactive exploration of citation networks with more user control. For example, adding multiple papers to a collection and then seeing common citations or references.

**Traditional indexes with AI features**: Traditional academic platforms such as Web of Science or Scopus now integrate AI to suggest related articles beyond simple citation links using keywords or topics). Google Scholar does a basic version (“Related articles” link); though it is not AI per se, it is algorithmic.

**NotebooksLM**: This product is excellent for connecting and summarizing papers, but the user must first upload or link the papers. It has many other features, such as the capacity to generate a podcast from one study or several studies and to allow chatting with a bunch of studies at once.

The benefit of connector tools is the capacity to find relevant research that keyword searches miss, because the terminology differs. Especially in interdisciplinary research, two papers might discuss similar concepts with different jargons; a connector tool that notices they cite a common third paper, for example, can bridge that gap for you.

**Figure 2.**
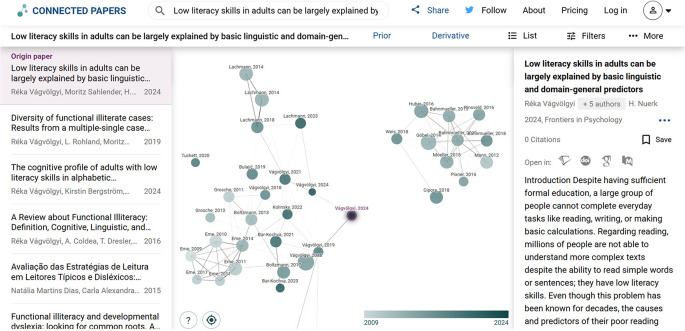
An AI “Connector” Tool in Action. Screenshot of a Connected Paper graph from the web-based interface of the tool (May 11 ^th^, 2025), generated for the original paper (at the center). Each node is a paper, and the edges (lines) indicate connections based on citation patterns and topic similarity. Such visual tools help to identify clusters of research and pivotal works at a glance, complementing traditional searches.

New tools provide systematic review functions using AI (e.g., DistillerSR or LaserAI), but we are unaware of any of these that are free for the user and have not been tested. It is clear, however, that automated systematic reviews will soon become the norm; more, we cannot predict. ^82^

### 4.3 Analysis and Data Support

General-purpose Gen AI can perform powerful calculations and show its work in formulas and proofs. As math is precise, it is easy for AI to become 100% accurate (zero error) in math cases [13] and very good in code generation for the most commonly used statistical software that are widely discussed on the Internet (e.g., Python and R are great, Stata and SPSS are not as good).

**Power Analysis**: In our combined experience, with decent prompting (see section 4), Gen-AI will make accurate calculations of the sample size, alpha, detectable effect size, and more, based on research design inputs. This generates the equations used to calculate the results.

**Data Extraction**: Gen AI often has access to most databases, such as the OECD or Eurostat, and can extract and prepare data. Data can be converted into almost any format for downloading. This is the case for ChatGPT and Gemini, and we assume other Gen AI are similar. Gen AI can also extract structured data from text. Although Gen AI output is not always reproducible given customizations, different computing environments, and proprietary architectures, it is possible to engineer prompts or *promptbooks* in a way that they become more reliable via testing and error minimization and are relatively reproducible. ^83^ Promptbooks serve as structured guides that catalog effective prompt patterns for various tasks. These codebooks compile tested prompt templates, strategies, and examples, enabling users to elicit desired outputs from AI models more efficiently. For example, unstructured descriptions of people in newspapers can be used to extract specific variables, such as age, sex, education, and occupation, using prompting (prompt book) techniques. ^84^

**Data Analysis:** We can upload data and ask Gen AI to execute the code to analyze the data. In this case, users should exercise caution. For example, ChatGPT can only run Python or R. Additionally, the user has little or no control over packages or versioning. The range of tasks generally includes everything Python and R can perform in the case of the former, which includes machine-learning applications. For example, Prandner et al. used ChatGPT to generate code and analyze data. ^85^ They discovered that Gen AI excelled at many but not all tasks and was poignantly better with open source and highly discussed software on the Web like R in comparison to others such as SPSS.

**Annotation:** Researchers have had great success in uploading text and asking Gen AI to qualitatively analyze and ‘code’ the text (what is known today as annotation). There is evidence that Gen AI can annotate as well as human coders in most cases ^86^ and often better, ^87^ ^,^ ^88^ but sometimes there are exceptional biases and systematically distorted errors, as seen in an example of coding interviews with displaced Rohinga. ^89^

**Fine-tuning **: Researchers seeking to train a machine-learning algorithm can ask Gen-AI to generate new sentences based on existing sentences to increase the size of the training data. These sentences can maintain the same meaning from a natural language processing perspective but reduce Type I and Type II error rates (higher F1, precision, and recall).

## 5. How to Interact with Gen AI: Prompting

Most mainstream Gen AI contain tools such as web searches and document analysis. Although there are differences in the training (the parameters of the artificial neural network) and power (GPU and server capacities) of the different Gen AIs, the real differences in performance come from the user. Gen AIs are highly sensitive to how users phrase their input, which is known as *prompting.* This section provides guidance on how to craft prompts to improve the utility of outputs.

At its core, a prompt is simply the input or question you provide to the AI model. However, writing a prompt is like writing the instructions for a very literal-minded, knowledgeable, but non-sentient assistant: You often need to be specific and clear about what you want, often in ways you do not need to be with a human. We have developed these definitions to guide users.

**Prompt**: Structured input comprising text, code, or other data designed to elicit a specific output from a Generative Artificial Intelligence (Gen AI) model by framing the task, constraints, and context.

**Prompting**: The practice of formulating and refining prompts to guide the model towards the desired outputs. In short, prompting is how we “program” a model with instructions every time we use it, without changing the model code or parameters.

If you want AI to help summarize an article, a prompt might be: *“Summarize the following article in one paragraph:”* followed by the article text. A more elaborate prompt may set the context and tone: *“You are an expert in the field. Summarize the following article in one paragraph, focusing on the main findings and their significance, in a neutral academic tone:”* followed by the text.

We categorize best prompting practices in the following four sections: 4.1. What you want, 4.2. How you want it, 4.3. tasks and examples, and 4.4. Interactive iteration.

### 5.1 What Do You Want?

Clearly specify the task that you want the AI to perform. The uncertainty expressed in a prompt is directly reflected as uncertainty in the AI answer. Simple prompts like, “describe social homophiliy” or “what is a regression” will lead to answers like what you would find on Wikipedia. Generalized, generic knowledge of the subject. If you tell the AI to “describe how social homophiliy impacts assortative mating in a country that has an ethnically diverse population and very high inequality,” the answer will be far more targeted and sophisticated. Assume that AI has nearly unlimited knowledge, but to tap into it, correct words must be used. Words and their ordering have meanings and linkages with other meanings.

Some common ideas of “what” to ask for are translation, summary, drafting (an email, report, social media post), comparing things (like articles or data), data analysis, internet search and interpretation, research design, filtering (removing unwanted things from text), reformatting (into certain styles), and rewriting (to have a more polite or academic tone). The desired output of AI is limited by our knowledge of what is possible for us and other human researchers. We are now in a completely new ‘ball game’ with Gen AI. Because AI is a trained artificial ‘brain,’ we could potentially get anything out of it, depending on the prompt.

With the help of Gen AI (here ChatGPT 4o), we came up with some research tasks that humans cannot do alone. These are *auto-synthesized interdisciplinary literature map.* This would most likely require an AI Agent, as the citations must be checked to ensure that they are not only accurate but also real. A *synthetic peer review panel* could prompt three different peer reviewers with different disciplines, expertise, and emotional states to critically review our paper. We could also ask AI to create theoretical functions that explain some phenomena in the world (see Online Appendix Prompt 1 [14] for an example of the likelihood of war).

### 5.2 How Do You Want It?

An effective prompt includes details of the desired format, length, style, and other output criteria. Currently, almost all Gen AI can output documents such as .docx, .pptx, images, .xlsx, .csv, .json, .md, .html and etc. [15]. For text, it can come in any style that a word processor can produce, for example, a list of bullet points, formulas, bold, italics, etc. The user can specify the style, for example, a certain academic style, such as the American Psychological Association, or in the style of a social media post or journalist. The font, spacing, and version of the language (Swiss vs. German) can all be specified. The tone can be adjusted and having the Gen AI ‘pretend’ it is a peer reviewer, critical boss, or emotionally sensitive colleague could all lead to more precise and useful outputs.

Specify the target audience if applicable. This will help an AI to adjust its complexity and use relevant information. For example, “Explain for a 12-year-old” versus “Provide a detailed technical explanation suitable for practicing researchers with at least a PhD in a social science discipline” would lead to very different outputs. For things that are really new or difficult for you, try asking “explain for a child,” and then adjusting the age upward; it can be incredibly helpful.

*Temperature* is a Gen AI-specific setting that ranges from 0 to 1. A high temperature (e.g., 0.8) yields more creative and varied outputs. Gen AI “understands” how to be literal or flowery and elaborate. A low temperature (e.g., 0.2) yields more focused and deterministic outputs. Setting this value to zero should result in the highest (and dryest) amount of factually consistent information. Most Gen AI will understand “Temperature 0.5”, but if not, you can implicitly guide this through wording (for example: “Give a straightforward, literal answer” vs “Feel free to be imaginative”).

It is important to understand that *every word* and *word combination* counts in shaping the output from Gen AI. By clearly outlining how you want the answer, you reduce the chance of getting a wall of text when you want a table or an overly casual answer when you want mathematical proof. Our human tendency to ask for things in a polite manner using “please” and “thank you” for example could actually lead to lower quality outputs [16]. ^90^ Gen AI will ‘think’ that it needs to formulate a polite response. Politeness from an NLP perspective means that it is acceptable to hide the truth somewhat to avoid upsetting the prompter. If you do not get what you want through prompting, think carefully about the word choice.

Most Gen AI have customization features. For example, in ChatGPT, one can click on the “Personalize” menu and set features that apply to every prompt. For example, one of us has as their settings the name the ChatGPT should call them, that they are an “Experienced scientific researcher” that the traits should be “Scientific, blunt, low temperature and using logic” and that they “use ChatGPT mostly for scientific writing, research and analysis.” This can save time, so a researcher does not always have to add these customizations to every prompt or chat.

### 5.3 Tasking with Examples

At the core of a prompt is a *task* that a Gen AI should undertake. The ideal prompt contains a single task, and everything in the prompt is related to that task. There are cases that require more than one task in a single prompt, and in these cases the tasks should be numbered or lettered, otherwise the different tasks ‘bleed’ into each other leading to mixed or undesired outcomes. It is beneficial to provide context, examples, or roles to get the most out of tasking.

Provide context. Gen AIs have no knowledge or memory of the world beyond what you give in the prompt, and their ANN is built on training data. If your question depends on a specific context (such as the passage of text, data, or the last thing you talked about in the conversation), make sure to include or reference it in the prompt. For example, instead of saying, ‘What does this result mean?’ (with no context), say “Given the results of the study (summarized as: XYZ), what does this result mean for practical applications in education?” Including a brief summary or the relevant excerpt in the prompt allows the model to base its answer on that rather than on general stored knowledge.

Provide examples. Examples are known as *shots* in computer science. You can sometimes save a lot of time trying to describe exactly what you want and how you want it by simply giving a few shots. You may want an abstract summarizing your work because you plan to submit it to a specific academic journal. You can give Gen AI a few abstracts from that journal and ask you to craft your abstract following the same style. However, we should keep in mind our strong caution regarding the use of AI-generated text. The output should be considered an example and rewritten in one’s own words.

Use role-playing. A popular prompting technique is to ask the model to “act as.” For example: “You are a statistician. Explain p-values in the style of a friendly tutor.” This sets a persona and can affect tone and content (making it more technical or accessible, depending on the role). This is useful for either leading the model to take on a certain voice or enforcing constraints. Another example: “You are an impartial research assistant that only uses evidence-based information. Answer the question …” Such role assignment can sometimes also bypass unhelpful general responses and obtain a more precise answer style.

### 5.4 Interactive Iteration

Prompting is not a single request. It is a dialog. If you do not obtain the desired results, refine your question. All Gen AI we are aware of operate inside of ‘chats.’ These are conversation-specific canals. Everything the AI responds to in each of these chats is based on the chat history. Normally, these chats are found by default on the left panel of a Gen AI user interface. Whether Gen AI saves the features of the chat or simply looks at the previous prompts in a conversation matters for the outcomes, and you might have to prompt it to read previous chats, especially if the chat history is very long or you have waited for a long time since last being active in that chat.

Chat channels significantly empower users. This means that you can set up what and how (see 5.1 and 5.2), and this is stored so that you do not need to keep asking. You could ask for a 200-word abstract in Modern Language Association (MLA) Author-Date format, and then a follow up would simply be a refinement, like “please remove sentence X, and add information about the case numbers” and you should again get a (roughly) 200-word abstract in MLA style by default from then on in that chat.

Simultaneously, it is important to start new chats for new prompt topics. If you ask about cats repeatedly in a chat history about how a correlation works and then ask about income inequality, you are likely to obtain information about income inequality correlations.

Prompting is iterative within chats, but can also be interactive across platforms. This is essentially what AI Agents like ‘Deep Research’ attempt to do with themselves (several ANNs running simultaneously), but you should also act as an agent seeking to get what you want (from several Gen Ais at once). For example, you might prompt several different Gen Ais (ChatGPT, DeepSeek, and Gemini) simultaneously and extract the most useful results. An agent creates a task list, attempts to execute it, and checks the results against various steps in the process, we find it helpful to keep in mind that both you and the Gen AI can and should do this actions for optimal results. When you do this it is known as “chain-of-thought” prompting, which calls on the Gen AI to do specific tasks in order that follow a logic or reasoning steps that mirror cognitive tasks. ^93^

To solidify these ideas, let us walk through an example of prompt evolution, as if we are “prompt engineers” [17] to refine our query:

Scenario: We want AI to help brainstorm research ideas on how generative AI can be used in adult education. We gave the naive prompt, “Give me research ideas for using AI in adult education.” AI likely gives a few generic ideas, likely surface-level, about AI tutors for adult learners or AI for personalized learning.

We think about how to improve the prompt: “I am looking for research project ideas on applications of generative AI in adult education. Please provide three detailed ideas, each a few sentences long, explaining the idea and why it is significant. Focus on practical use cases in adult learning settings (such as workplace training or continuing education), and make sure the ideas are distinct from each other.” Although this sounds like multiple tasks, they all relate to the core task of listing the three ideas. They fine-tune the task rather than give new tasks. This included several shots. AI should now produce a structured list of the three ideas, each with some details and explanations.

It is possible that these ideas are still somewhat common. We could add: “For each idea, mention any challenges or research questions that would need to be addressed (e.g., privacy, effectiveness, user engagement).” Here, we take advantage of the fact that chat history is stored. Therefore, we did not need to rewrite the second prompt. AI should now append or integrate challenges into each of the already listed ideas.

Now that we are satisfied with the ideas, we can ask it to produce a report with valid references. This is only possible with a literature database or an Internet search tool. We then prompt: “Name any existing studies or examples that relate to these ideas. Use an Internet search to ensure true, high-quality citations.” We can refine this request until satisfied and then ask for the final report: “Now combine the preceding into a final report with the following components: 1. a creative title, 2. an image on the cover page that captures advances in adult learning, and 3. One page devoted to each of the three project ideas with at least two related citations integrated per project (in author date format plus a link), 4. A paragraph summary at the end about how the three projects all would contribute to improving scientific knowledge in adult education research”. The results are presented in Online Appendix, Prompt 2 [18].

This iterative process shows how we can coax AI towards what we need. In each step, we provided more structures or asked for more depth. Finally, we have a finished product that appears as something we can submit as part of our work. However, in this example we would not do that and instead re-write the entire report, or write one from scratch based on the ideas, and then double check all logic and citations, as this is the best practice and allows us to have our own copyrightable work.

Moreover, looking at the results carefully in Appendix Prompt 2, there is more to do – *more to prompt* in other words. The image was taken from another source, and there may be issues with its use. When clicking on the source, the link does not lead to a website containing the image; therefore, it is unclear whether ChatGPT invented the image. The authors’ date citation request was ignored, perhaps because we were also asked to provide links. Refining these steps would require more iterative prompts. We could also ask for it in the PDF format once we have something better. We have intentionally left this prompting dialog unfinished, to keep users fully aware that without careful checking there are likely mistakes, omissions or errors in Gen AI output.

Prompting is a skill that develops through experimentation. Often, reading what others have done. There are many prompting videos and resources available online. A prompt that works in one context might not directly transfer to another, always thinking about the principles rather than rigid templates.

In teaching or working with colleagues, it can be useful to show how much a prompt can change output quality. For example, giving a poorly specified task versus a well-crafted prompt and comparing the results often convinces people of why prompting matters. In prompting you not just to ask a question, you are guiding a process.

## 8. Conclusion

Artificial intelligence transforms how we find information, analyze data, and generate content. For researchers, teachers, and academic staff, the promise of AI in speeding up literature reviews, developing course materials, translation, offering writing assistance, and uncovering patterns in data is fantastic. At the same time, there are pitfalls and great uncertainties regarding when and how to use AI. Combining our practical experience as both scholars and regular users of Gen AI with a broad range of informational sources this semi-formal text provided an introduction to how AI works, what is best to do with it, what tools are available and some tips on prompting.

A crucial lesson is that AI is a tool and not an oracle. AI’s knowledge is based on training data and pattern recognition, not on true comprehension or authority. It can err confidently. One should *trust but verify.* This follows ideal scientific practice recommended long before AI by Robert K. Merton. ^94^ In particular, “organized skepticism” should be our collective position on AI outputs. Knowing the basics of artificial neural networks trained on massive amounts of data, should help bolster this skepticism as anything generated in an opaque box is inconsistent with open science – intransparent and irreproducible. ^54^ ^,^ ^55^ ^,^ ^95^

The fact that AI is not an oracle, but can behave like one, is dangerous. We may become confused and think that AI is telling us the truth when instead, it maximizes our desires as based on our prompts. This introduces the threat of scientific AI hacking. A researcher might keep prompting until they get the answer they want, rather than one that is useful and reliable. AI can also be used to p-hack directly, dredging through data to find unique but not generalizable associations that a researcher can then pretend to have ‘discovered’ in a well-designed hypothesis test. Demystifying why AI might give an incorrect answer, be biased, or even used intentionally to hack should be a core task of learning for academics today.

Understanding the limitations of AI, such as a lack of up-to-date knowledge or inability to reason like a human, empowers users to avoid misusing AI. It is good to offload cognitive burdens, but this should be done in a manner where the human user retains control over every step in the workflow: That they understand (check, vet, interrogate) everything that is being generated. For example, if you do not know how to use R statistical software, then having AI write R code for your research might be a bad idea. We believe that it is the task of researchers to focus on how AI can enhance their work, while recognizing that their work will be best when they also understand the underlying mechanisms (methods, theories, literature) of their craft. AI extends and augments what a researcher can do; it is not a replacement for researchers.

Recent and rapidly changing trends shape how researchers use AI tools [19]. Academic publishers and databases have also incorporated AI. For example, Springer experimented with GPT-based assistants on their platforms to help find content. Zotero, an open-source, non-profit reference manager, now has a plugin (“ZoteroGPT”) that can summarize PDFs in your library. We generated a podcast from this study using NotebookLM, which is highly accurate [20]. Because of these technologies, Gen AI will revolutionize science communication. It can summarize the mountains of research in a way that general audiences of even school children will understand, but the risk of hallucination and misinformation is ever present, and the rate of adoption is so high with Gen AI that there will be no way to vet all these communications. ^96^ For example, Robert F. Kennedy Jr. produced a report on children’s health reports generated from Gen AI with errors and fake citations. ^97^

Science policy organizations encourage the exploration of AI in research. In Germany, initiatives within the National Research Data Infrastructure (NFDI) and in the U.S. within the National Institute of Standards and Technology (NIST) have investigated how AI can help manage and analyze research data. Data management plans are beginning to include sections on AI usage. This means that support for researchers may improve, for example, institutional access to better AI tools that are privacy-compliant, such as Microsoft’s Copilot (provided your institution trusts Microsoft).

Using Gen AI to produce knowledge that is then used to populate academic and web-based sources (like Wikipedia) carries long-term risks because it can lead to what computer scientists call *model collapse.* This is a phenomenon where the data used to train and fine-tune new Gen AI systems is more and more a product of Gen AI rather than humans. This can cause the knowledge and practical functions of AI to collapse because semantic meaning is no longer human-based, but AI based – the implications of which are unknown. This means that human knowledge may find itself in a non-recursive feedback loop over time, and its utility to human realities and social worlds collapses with it.

New AI-driven platforms for scholarly collaboration are emerging as tools that automatically write the first draft of a literature review section based on a shared library of papers that a team can then edit. These blur the line between source finding and writing. While promising for productivity, they again require that researchers do not skip the understanding part: an AI-generated literature review is only as good as the sources it was given, and cannot interpret significance or context the way a human researcher can.

AI has become an indispensable assistant in the handling of information. By learning what different tools can be offered, researchers can significantly enhance their efficiency. However, the effective use of these tools involves knowing their scope and limits. As the saying goes among statistical modelers: ‘garbage in, garbage out’. This applies to the quality of the AI augmented research process. It depends on the quality of your questions, data sources, training data, and, ultimately, your own judgment. Again, we remind the reader that they are responsible for everything on which they put their names. We cannot stress enough that copying and pasting AI written work into our scholarly works is a dangerous idea and should be avoided at all costs. When using AI-generated text, read every word, and (re-)write your own words to describe what you want.

## Author contributions statement

Conceptualization: NB, HHVN; Data Curation: N/A; Formal Analysis: N/A; Funding Acquisition: NB; Investigation: NB, HHVN; Methodology: NB, HHVN; Project Administration: NB; Resources: NB, HHVN; Software: NB, HHVN; Supervision: NB; Validation: N/A; Visualization: NB, HHVN; Writing Original Draft: NB, HHVN; Writing Review and Editing: NB.

## Data availability statement

No data are associated with this article. All prompts and outputs used by Gen AI are available in the footnotes and appendix of this paper.

## Footnotes

1
It is perhaps not entirely a coincidence “GPT” is in the name “ChatGPT”. Although this technically refers to “Generative Pre-trained Transformer” it cleverly foreshadows Gen AI’s role in human existence.

2
Online Appendix: https://osf.io/xf52m/ Glossary: https://osf.io/pkec5

3
See for example: https://en.wikipedia.org/wiki/Neural_network_(machine_learning)

4

https://osf.io/fguk6

5
The number of layers, learning rate, optimization and regularization which are known as *hyperparameters* and are set prior to training, unlike parameters which are adjusted and updated during training.

6

https://moebio.com/mind/

7
Computer scientists experimented with hybrid models in developing Gen AI systems but quickly discovered that the encoder portion was not necessary. It consumed time and resources without improving the results.

8

https://agents4science.stanford.edu/

9
A German idiom meaning “egg-laying woolly milk sow,” a mythical creature that provides eggs, wool, milk, and meat – in other words, a single source of all needs.

10
Deutsche Forschungsgemeinschaft in German.

11
Although beyond our scope here it is worth mentioning that there are a watershed of tools to help students study such as Alice AI, Mindgrasp, Quizlet, Studley AI, Wolfram Alpha and many more.

12
Grok is also a popular Gen AI developed by xAI, an American company. Launched in November 2023, Grok is integrated with the social media platform X (formerly Twitter). It can be tailored to specifications, but is designed initially to be wittier, mimicking social media behavior. It is gaining rapidly in popularity, but we lack evidence to date of wide usage for academic purposes.

13
The word “nearly” is crucial here. There are now infamous examples of Gen AI getting specific mathematical calculations wrong. Even after running Python code to get the correct answer, but still insisting their wrong answer is correct. See for example https://i.redd.it/ii5bfbplvf2f1.jpeg. However, look at our example asking to generate the formulas that build a slopes-as-outcomes regression model, they are flawless https://chatgpt.com/share/e/684af1ce-c398-8004-b2b3-6816601648c7.

14

https://osf.io/fguk6

15
Note that output follow proprietary conventions. This means that Gemini will not generate a Word document and instead offers a Google Doc.

16
Use of these words could also increase the usage of computational resources ^91^; however, there are reasons to remain polite in Human-AI interaction to avoid degradation of Human-Human kindness in society. ^92^

17
We are not particular fans of the term “prompt engineer” as it seems to be a buzzword. We do not see prompting as a skill that requires special job titles. Analogous would be “internet search engineer” which at this point in time sounds absurd as a job title.

18

https://osf.io/fguk6

19
And as things are rapidly evolving, there are meta-sources to help us find AIs for specific tasks like “There’s An AI For That” (TAAFT) https://theresanaiforthat.com/.

20

https://notebooklm.google.com/notebook/42571778-12bb-41a1-b6a6-0d52c114d301/audio
