# Supplementary material for: An Introduction to Generative Artificial Intelligence for Academics
Source: F1000Res. 2026 Feb 16;14:655. Originally published 2025 Jul 4. [Version 2] doi: 10.12688/f1000research.166513.2 (PMC13229007; doi:10.12688/f1000research.166513.2)
Supplement: Peer review history

# Reviewer report: Reviewer response for version 2

**DOI:** 10.5256/f1000research.194771.r459043

**Published:** 2026-02-25

**Authors:** Kezia Mkwizu

**Affiliations:** 1 Independent Researcher, Dar es Salaam, Tanzania

The revisions made to the article are satisfactory.

Is the review written in accessible language?

Yes

Are all factual statements correct and adequately supported by citations?

Partly

Are the conclusions drawn appropriate in the context of the current research literature?

Partly

Is the topic of the review discussed comprehensively in the context of the current literature?

Partly

Reviewer Expertise:

NA

I confirm that I have read this submission and believe that I have an appropriate level of expertise to confirm that it is of an acceptable scientific standard.

# Reviewer report: Reviewer response for version 2

**DOI:** 10.5256/f1000research.194771.r459042

**Published:** 2026-02-18

**Authors:** William J Marin-Rodriguez

**Affiliations:** 1 Universidad Nacional José Faustino Sánchez Carrión, Huacho, Peru

The authors addressed the comments made on the document.

Is the review written in accessible language?

Yes

Are all factual statements correct and adequately supported by citations?

Partly

Are the conclusions drawn appropriate in the context of the current research literature?

Yes

Is the topic of the review discussed comprehensively in the context of the current literature?

Partly

Reviewer Expertise:

Machine learning, IA, IT

I confirm that I have read this submission and believe that I have an appropriate level of expertise to confirm that it is of an acceptable scientific standard.

# Reviewer report: Reviewer response for version 1

**DOI:** 10.5256/f1000research.183507.r417098

**Published:** 2025-10-14

**Authors:** Kezia Mkwizu

**Affiliations:** 1 Independent Researcher, Dar es Salaam, Tanzania

The general overview is interesting and provides insights on Gen AI for academics. Some areas need improvements as highlighted in the comments below.  

 A: The topic is important, and the review discussed is comprehensive in the context of the current literature since citations up to year 2025 have been cited. This is good. Please see comments to improve the writing and discussion of the general review:

***Comment No. 1***

 Whilst the introduction section mentions the approach of using “a semi-informal and following a structure of a talk or course”, this was not highlighted in the abstract. Therefore, the abstract lacks a methodology approach. The Author(s) may wish to include this so that the abstract has a methods component to it.

***Comment No. 2***

 Below paragraph is informative but lacks citations. It would be good to support this discussion paragraph with citations.

 “Gen AI’s ability to appear knowledgeable is a direct function of the data it has seen. If trained on a large database of images or corpora of text, it internalizes an enormous range of facts and associations, such as in a human brain’s neural network. An artificial neural network does not have an active understanding or fact-checking mechanism beyond the patterns learned in the data. As a result, AI knowledge is inherently statistical; it knows which words or features tend to go together in the training data, not whether statements are objectively true. This has implications discussed later, such as the risk of confidently stated but false outputs. When Gen AI outputs are very far from the truth or the target of the prompt, they are known as hallucinations. We do not know exactly how a Gen AI hallucinates, but with millions of mathematical equations activated simultaneously, it is perhaps not surprising.”

***Comment No. 3***

 The Author(s) can also add more literature as suggested below which can help to strengthen the discussion of the review including the issue of practices. Please see below links of suggested literature;

https://academic.oup.com/nsr/article/12/5/nwaf050/8029900 (Ref 1)

https://www.sciencedirect.com/science/article/pii/S0160791X2500003X (Ref 2)

https://www.emerald.com/ijilt/article-abstract/41/2/180/1215652/How-generative-artificial-intelligence-has-blurred?redirectedFrom=fulltext (Ref 3)

https://educationaltechnologyjournal.springeropen.com/articles/10.1186/s41239-024-00453-6 (Ref 4)

https://www.mdpi.com/2227-7102/15/3/343 (Ref 5)

 B: On the factual statements and adequate support by citations is generally good but some, sentences/lines and words like “backpropagation” need more literature to back it up.

***Comment No. 1***

 On page 3 of 22: the below sentence is great. But it would help to have a citation so that reader(s) can know where the word “ backpropagation” was sourced from.

 “This is an algorithmic process known as backpropagation.”

 Examples of suggested literature can be as per below links:

https://www.mdpi.com/2079-9292/11/10/1590 (Ref 6)

https://www.mdpi.com/2409-9287/7/2/36 (Ref 7)​​​​​​​

https://www.nature.com/articles/s41467-024-53827-9  (Ref 8)​​​​​​​

***Comment No. 2***

 On page 6 of 22, the reader(s) by now know what ANNs is since you have explained this and abbreviated it well. For consistency, you may correct below line;

 … multiple artificial neural networks …

 to just read as:

 … multiple ANNs …

 Correct also for page 7 of 22 and other pages of the manuscript. For example, on page 7 of 22, the line below:

 “Artificial neural networks have so many nodes and layers that …”

 Can be improved to read as:

 “The ANNs have so many nodes and layers that …”

***Comment No. 3***

 Table 1 is good but has no sources. Where was this information sourced from? Citations are needed here and should also be included in the reference list.

***Comment No. 4***

 Figure 2 is helpful but has no source. Please include source so that reader(s) are well informed.

***Comment No. 5***

 On page 12 of 22, the information for examples of (Connected Paper, Local Citation Network, Inciteful and Research Rabbit, Traditional indexes with AI features, NotebooksLM) have no citations. Please show citations so that reader(s) are informed of the sources for these examples.

  C: The conclusion can improve and please see comments

***Comment No. 1***

 The abstract has no conclusion. It will be better for the Author(s) to improve the abstract with what the conclusion of this primer cover is. A brief sentence will help.

***Comment No.2***

 For the conclusion section from page 16 of 22 to 17 of 22, it would help for the Author(s) to create a subheading on the implications of this general overview. The reader(s) would be interested to know the implications from this general overview. Is it a managerial implication or is it a practical implication?

***Comment No. 3***

 It is very good that the first sentence of the abstract mentions the review is for academics, students, workers, researchers and teachers. However, in the conclusion section there is no information in reference to students. There is a bit of mismatch here and no consistency. The Author(s) are advised to show what this review has discussed in reference to students as well as show it in the conclusion section.

Is the review written in accessible language?

Yes

Are all factual statements correct and adequately supported by citations?

Partly

Are the conclusions drawn appropriate in the context of the current research literature?

Partly

Is the topic of the review discussed comprehensively in the context of the current literature?

Partly

Reviewer Expertise:

Tourism, higher education

I confirm that I have read this submission and believe that I have an appropriate level of expertise to confirm that it is of an acceptable scientific standard, however I have significant reservations, as outlined above.

## References

[1] : Generative artificial intelligence: a historical perspective. *National Science Review* .2025;12(5) : 10.1093/nsr/nwaf050 10.1093/nsr/nwaf050 PMC1197024540191253

[2] : Generative Artificial Intelligence (GenAI) in the research process – A survey of researchers’ practices and perceptions. *Technology in Society* .2025;81: 10.1016/j.techsoc.2025.102813 10.1016/j.techsoc.2025.102813

[3] : How generative artificial intelligence has blurred notions of authorial identity and academic norms in higher education, necessitating clear university usage policies. *The International Journal of Information and Learning Technology* .2024;41(2) : 10.1108/IJILT-11-2023-0213 180-193 10.1108/IJILT-11-2023-0213

[4] : Generative AI and the future of higher education: a threat to academic integrity or reformation? Evidence from multicultural perspectives. *International Journal of Educational Technology in Higher Education* .2024;21(1) : 10.1186/s41239-024-00453-6 10.1186/s41239-024-00453-6

[5] : The Impact of Artificial Intelligence (AI) on Students’ Academic Development. *Education Sciences* .2025;15(3) : 10.3390/educsci15030343 10.3390/educsci15030343

[6] : Comprehensive Overview of Backpropagation Algorithm for Digital Image Denoising. *Electronics* .2022;11(10) : 10.3390/electronics11101590 10.3390/electronics11101590

[7] : Backpropagation of Spirit: Hegelian Recollection and Human-A.I. Abductive Communities. *Philosophies* .2022;7(2) : 10.3390/philosophies7020036 10.3390/philosophies7020036

[8] : The backpropagation algorithm implemented on spiking neuromorphic hardware. *Nature Communications* .2024;15(1) : 10.1038/s41467-024-53827-9 10.1038/s41467-024-53827-9 PMC1154937839516210

# Response

**Published:** 2026-01-03

**Authors:** Nate Breznau

Response Letter:

 Thank you for the opportunity to revise our manuscript. We address all reviewer comments in-line in the following.

**Reviewer 2:**

 [COMMENT] Whilst the introduction section mentions the approach of using “a semi-informal and following a structure of a talk or course”, this was not highlighted in the abstract. Therefore, the abstract lacks a methodology approach. The Author(s) may wish to include this so that the abstract has a methods component to it.[HN4] 

 [OUR RESPONSE] Good idea. We have added a sentence to the abstract to inform the reader of this in advance.

***Comment No. 2***

 [COMMENT] Below paragraph is informative but lacks citations. It would be good to support this discussion paragraph with citations.

 “Gen AI’s ability to appear knowledgeable is a direct function of the data it has seen. If trained on a large database of images or corpora of text, it internalizes an enormous range of facts and associations, such as in a human brain’s neural network. An artificial neural network does not have an active understanding or fact-checking mechanism beyond the patterns learned in the data. As a result, AI knowledge is inherently statistical; it knows which words or features tend to go together in the training data, not whether statements are objectively true. This has implications discussed later, such as the risk of confidently stated but false outputs. When Gen AI outputs are very far from the truth o the target of the prompt, they are known as hallucinations. We do not know exactly how a Gen AI hallucinates, but with millions of mathematical equations activated simultaneously, it is perhaps not surprising.”

 [OUR RESPONSE] We have now revised the paragraph and added references, so that readers can immediately find them (rather than look for them in the next section as it was before).

 [HN5] 

***Comment No. 3***

 [COMMENT] The Author(s) can also add more literature as suggested below which can help to strengthen the discussion of the review including the issue of practices. Please see … suggested literature;

 [OUR RESPONSE] We have added the citations suggested and more on “backpropagation”, thank you so much for pushing us to do this. The paper is much stronger now.

***Comment No. 1***

 [COMMENT] On page 3 of 22: the below sentence is great. But it would help to have a citation so that reader(s) can know where the word “ backpropagation[HN6] ” was sourced from.

 “This is an algorithmic process known as backpropagation.”

 [OUR RESPONSE] We added the suggested literature and/or sought out even more specific/deeper citations to our exact points. Thanks.

***Comment No. 2***

 [COMMENT] On page 6 of 22, the reader(s) by now know what ANNs is since you have explained this and abbreviated it well. For consistency, you may correct below line;

 … multiple artificial neural networks …

 to just read as:

 … multiple ANNs …

 Correct also for page 7 of 22 and other pages of the manuscript. For example, on page 7 of 22, the line below:

 “Artificial neural networks have so many nodes and layers that …”

 Can be improved to read as:

 “The ANNs have so many nodes and layers that …”[HN7] 

 [OUR RESPONSE] Very helpful suggestions! We have edited the text accordingly.

***Comment No. 3***

 [COMMENT] Table 1 is good but has no sources. Where was this information sourced from? Citations are needed here and should also be included in the reference list.[HN8] 

 [OUR RESPONSE] It is difficult to get exact usage statistics, as companies do not share this private data, or at least not in any comparable format. We have cited two sources, but we otherwise relied on our own expertise and now stated this in the text.

***Comment No. 4***

 [COMMENT] Figure 2 is helpful but has no source. Please include source so that reader(s) are well informed.

 [OUR RESPONSE] We now clarify this in the summary of the Figure – it is a screenshot from the App.

***Comment No. 5***

 [COMMENT] On page 12 of 22, the information for examples of (Connected Paper, Local Citation Network, Inciteful and Research Rabbit, Traditional indexes with AI features, NotebooksLM) [HN9] have no citations. Please show citations so that reader(s) are informed of the sources for these examples.

 [OUR RESPONSE] We have no citations to offer here, and are not sure what the reviewer is asking for. All of these tools are easy to find on the internet. We could provide the URLs but this seems unnecessary.

  C: The conclusion can improve and please see comments

***Comment No. 1***

 [COMMENT] The abstract has no conclusion. It will be better for the Author(s) to improve the abstract with what the conclusion of this primer cover is. A brief sentence will help.[HN10] 

 [OUR RESPONSE] We have added a sentence on the purpose of the paper to be a primer on Gen AI. As this is not a research paper, we think this is a conclusion in a way. That the researcher will learn basics of Gen AI in our paper.

***Comment No.2***

 [COMMENT] For the conclusion section from page 16 of 22 to 17 of 22, it would help for the Author(s) to create a subheading on the implications of this general overview. The reader(s) would be interested to know the implications from this general overview. Is it a managerial implication or is it a practical implication?

 [OUR RESPONSE] This is a good idea, thanks. We do not have enough to write an entire section; however, we added this sentence to the Conclusion and hope it clarifies that this is a practical application “Combining our practical experience as both scholars and regular users of Gen AI with a broad range of informational sources this semi-formal text has practical implications in helping those in academia – students, faculty and staff – to be informed and improve their work.”

***Comment No. 3***

 [COMMENT] It is very good that the first sentence of the abstract mentions the review is for academics, students, workers, researchers and teachers. However, in the conclusion section there is no information in reference to students. There is a bit of mismatch here and no consistency. The Author(s) are advised to show what this review has discussed in reference to students as well as show it in the conclusion section.

 [OUR RESPONSE] We agree and already the new sentence in the Conclusion (mentioned in the previous response) mentions students directly, and we added the following sentence “This is particularly relevant for students, as they use Gen AI at the highest rates, are the least skeptical of its risks and in a phase of critical thinking development which AI might blunt”

  [HN4]To be edited at the end

  [HN5]fixed

  [HN6]fixed

  [HN7]fixed

  [HN8]Do we cite the models or? I’m not sure what kind of citations they want.

[HN10]To be edited at the end

# Reviewer report: Reviewer response for version 1

**DOI:** 10.5256/f1000research.183507.r397725

**Published:** 2025-07-31

**Authors:** William J Marin-Rodriguez

**Affiliations:** 1 Universidad Nacional José Faustino Sánchez Carrión, Huacho, Peru

**1. Is the topic of the review discussed comprehensively in the context of the current literature?**

**Answer: Partly**

 The article does an excellent job of covering a wide range of relevant and current topics for an introductory piece. It cites recent developments (up to 2025) and includes discussions on very current tools and controversies. The selection of literature is appropriate for an introductory guide, mixing academic sources with news articles and online resources that reflect the field's rapid and discursive development.

**Constructive Feedback:**

- **Deepen the Ethics Section:** The ethics section is solid but could be enhanced by a deeper connection to foundational literature in AI ethics. While the "neutrality thesis" is mentioned, a brief discussion of more established ethical frameworks (e.g., principles of fairness, accountability, and transparency) would strengthen the arguments.
- **Contextualize Tool Selection:** A useful list of tools is presented, but it would be even more valuable to readers if the authors briefly explained the criteria for their selection. Why these tools and not others? A short justification would strengthen this section.

**2. Are all factual statements correct and adequately supported by citations?**

**Answer: Partly**

 Overall, the article presents accurate and well-supported factual information, especially when explaining technical concepts accessibly and referring to institutional guidelines and well-known cases. The explanations of how ANNs and LLMs work are correct for the target audience's level.

**Points that *must* be addressed to make the article scientifically sound:**

- **Factual Error in a Key Citation:** There is a significant inconsistency in the conclusion. The text states: "For example, Robert F. Kennedy Jr. produced a report on children's health generated from Gen AI with errors and fake citations.52". However, reference 52 points to a
*Science Insider* article titled: "Trump officials downplay fake citations in high-profile report on children's health". These appear to be two different incidents attributed to the same source. It is crucial that the authors verify this claim and cite it correctly. This type of error can undermine the credibility of the manuscript.

**Constructive Feedback:**

- **Citations for Practical Claims:** Some practical claims, such as the idea that politeness in prompts could lead to lower-quality outputs, are based on a news article. While interesting, the claim would be stronger if framed as an anecdotal observation or linked to research in natural language processing, if available.

**3. Is the review written in accessible language?**

**Answer: Yes**

 The authors have successfully achieved their goal of creating a "less technical introduction". The language is clear, direct, and avoids unnecessary jargon. When technical terms (e.g., "backpropagation," "tokens") are introduced, they are explained in a simple and intuitive manner. The semi-informal tone and use of analogies (like comparing network weights to regression coefficients) make the content highly digestible for academics from diverse disciplines. The structure is logical and effectively guides the reader from basic concepts to practical applications.

**4. Are the conclusions drawn appropriate in the context of the current research literature?**

**Answer: Yes**

 The article's conclusions are balanced, thoughtful, and highly appropriate. The core message—that AI is a supportive tool demanding critical skepticism, human oversight, and responsibility—aligns perfectly with the emerging consensus in the academic literature and the institutional guidelines cited. The warnings about risks such as "scientific Al hacking," the perpetuation of bias, and cognitive offloading are timely and well-grounded in the arguments presented throughout the text. The conclusions offer a pragmatic and responsible perspective that will be of great utility to the target audience.

**Publication Decision**

**Decision: Minor Revisions**

**Justification:** This article is a valuable, well-written, and timely contribution to an important topic. The authors have succeeded in creating an accessible and practical guide for a non-specialist academic audience. The structure is clear, the tone is appropriate, and the scope of the review is well-suited for an introduction.

 The decision for "Minor Revisions" is based on the fact that the required changes are not extensive but are absolutely critical for the paper's scientific integrity. The primary mandatory revision is the correction of a significant factual error in a citation within the conclusion. While other suggestions are constructive, this point is non-negotiable.

 Once this correction is made, along with consideration of the other minor suggestions, the paper will be a strong and publishable manuscript. It has the potential to be a highly cited and useful resource for the academic community navigating the complexities of generative AI.

Is the review written in accessible language?

Yes

Are all factual statements correct and adequately supported by citations?

Partly

Are the conclusions drawn appropriate in the context of the current research literature?

Yes

Is the topic of the review discussed comprehensively in the context of the current literature?

Partly

Reviewer Expertise:

Machine learning, IA, IT

I confirm that I have read this submission and believe that I have an appropriate level of expertise to confirm that it is of an acceptable scientific standard, however I have significant reservations, as outlined above.

# Response

**Published:** 2026-01-03

**Authors:** Nate Breznau

Thank you for the opportunity to revise our manuscript. We address all reviewer comments in-line in the following.

**Reviewer 1:**

**[COMMENT]** The ethics section is solid but could be enhanced by a deeper connection to foundational literature in AI ethics. While the "neutrality thesis" is mentioned, a brief discussion of more established ethical frameworks (e.g., principles of fairness, accountability, and transparency) would strengthen the arguments.

**[OUR RESPONSE]**

- **Contextualize Tool Selection:** A useful list of tools is presented, but it would be even more valuable to readers if the authors briefly explained the criteria for their selection. Why these tools and not others? A short justification would strengthen this section.[HN1] 

**[COMMENT] **There is a significant inconsistency in the conclusion. The text states: "For example, Robert F. Kennedy Jr. produced a report on children's health generated from Gen AI with errors and fake citations.52". However, reference 52 points to a[HN2] *Science Insider* article titled: "Trump officials downplay fake citations in high-profile report on children's health". These appear to be two different incidents attributed to the same source. It is crucial that the authors verify this claim and cite it correctly. This type of error can undermine the credibility of the manuscript.

**[OUR RESPONSE].** The article we cite “Trump officials downplay fake citations in high-profile report on children's health” ( https://www.science.org/content/article/trump-officials-downplay-fake-citations-high-profile-report-children-s-health), is about this report which was produced (spearheaded) by Robert F. Kenndy Jr. We selected the article because of this event (as we were watching it on the news as it happened). I’m not sure where the misunderstanding is, but I encourage the reviewer to look at the article again and double check because it provides evidence that this event took place! If we made a mistake, we will correct it, but it looks accurate to us.

**[COMMENT]** Some practical claims, such as the idea that politeness in prompts could lead to lower-quality outputs, are based on a news article. While interesting, the claim would be stronger if framed as an anecdotal observation or linked to research in natural language processing, if available.[HN3] 

**[OUR RESPONSE]** This is a good point. We added a citation to a study on this now in the main text and a blog post is cited in the footnote discussing why kindness might be good for society. 

  [HN1]This could be useful: LLM Leaderboard - Comparison of over 100 AI models from OpenAI, Google, DeepSeek & others | Artificial Analysis

 Also this: AI | 2025 Stack Overflow Developer Survey

  [HN2] perhaps you cited the wrong article?

[HN3]This paper is relevant: Should We Respect LLMs? A Cross-Lingual Study on the Influence of Prompt Politeness on LLM Performance - ACL Anthology
