# Supplementary material for: An Introduction to Generative Artificial Intelligence for Academics
Source: F1000Res. 2025 Jul 4;14:655. [Version 1] doi: 10.12688/f1000research.166513.1 (PMC13229007; doi:10.12688/f1000research.166513.1)
Supplement: Peer review history

# Response

**Published:** 2026-01-03

**Authors:** Nate Breznau

Response Letter:

 Thank you for the opportunity to revise our manuscript. We address all reviewer comments in-line in the following.

**Reviewer 2:**

 [COMMENT] Whilst the introduction section mentions the approach of using “a semi-informal and following a structure of a talk or course”, this was not highlighted in the abstract. Therefore, the abstract lacks a methodology approach. The Author(s) may wish to include this so that the abstract has a methods component to it.[HN4] 

 [OUR RESPONSE] Good idea. We have added a sentence to the abstract to inform the reader of this in advance.

***Comment No. 2***

 [COMMENT] Below paragraph is informative but lacks citations. It would be good to support this discussion paragraph with citations.

 “Gen AI’s ability to appear knowledgeable is a direct function of the data it has seen. If trained on a large database of images or corpora of text, it internalizes an enormous range of facts and associations, such as in a human brain’s neural network. An artificial neural network does not have an active understanding or fact-checking mechanism beyond the patterns learned in the data. As a result, AI knowledge is inherently statistical; it knows which words or features tend to go together in the training data, not whether statements are objectively true. This has implications discussed later, such as the risk of confidently stated but false outputs. When Gen AI outputs are very far from the truth o the target of the prompt, they are known as hallucinations. We do not know exactly how a Gen AI hallucinates, but with millions of mathematical equations activated simultaneously, it is perhaps not surprising.”

 [OUR RESPONSE] We have now revised the paragraph and added references, so that readers can immediately find them (rather than look for them in the next section as it was before).

 [HN5] 

***Comment No. 3***

 [COMMENT] The Author(s) can also add more literature as suggested below which can help to strengthen the discussion of the review including the issue of practices. Please see … suggested literature;

 [OUR RESPONSE] We have added the citations suggested and more on “backpropagation”, thank you so much for pushing us to do this. The paper is much stronger now.

***Comment No. 1***

 [COMMENT] On page 3 of 22: the below sentence is great. But it would help to have a citation so that reader(s) can know where the word “ backpropagation[HN6] ” was sourced from.

 “This is an algorithmic process known as backpropagation.”

 [OUR RESPONSE] We added the suggested literature and/or sought out even more specific/deeper citations to our exact points. Thanks.

***Comment No. 2***

 [COMMENT] On page 6 of 22, the reader(s) by now know what ANNs is since you have explained this and abbreviated it well. For consistency, you may correct below line;

 … multiple artificial neural networks …

 to just read as:

 … multiple ANNs …

 Correct also for page 7 of 22 and other pages of the manuscript. For example, on page 7 of 22, the line below:

 “Artificial neural networks have so many nodes and layers that …”

 Can be improved to read as:

 “The ANNs have so many nodes and layers that …”[HN7] 

 [OUR RESPONSE] Very helpful suggestions! We have edited the text accordingly.

***Comment No. 3***

 [COMMENT] Table 1 is good but has no sources. Where was this information sourced from? Citations are needed here and should also be included in the reference list.[HN8] 

 [OUR RESPONSE] It is difficult to get exact usage statistics, as companies do not share this private data, or at least not in any comparable format. We have cited two sources, but we otherwise relied on our own expertise and now stated this in the text.

***Comment No. 4***

 [COMMENT] Figure 2 is helpful but has no source. Please include source so that reader(s) are well informed.

 [OUR RESPONSE] We now clarify this in the summary of the Figure – it is a screenshot from the App.

***Comment No. 5***

 [COMMENT] On page 12 of 22, the information for examples of (Connected Paper, Local Citation Network, Inciteful and Research Rabbit, Traditional indexes with AI features, NotebooksLM) [HN9] have no citations. Please show citations so that reader(s) are informed of the sources for these examples.

 [OUR RESPONSE] We have no citations to offer here, and are not sure what the reviewer is asking for. All of these tools are easy to find on the internet. We could provide the URLs but this seems unnecessary.

  C: The conclusion can improve and please see comments

***Comment No. 1***

 [COMMENT] The abstract has no conclusion. It will be better for the Author(s) to improve the abstract with what the conclusion of this primer cover is. A brief sentence will help.[HN10] 

 [OUR RESPONSE] We have added a sentence on the purpose of the paper to be a primer on Gen AI. As this is not a research paper, we think this is a conclusion in a way. That the researcher will learn basics of Gen AI in our paper.

***Comment No.2***

 [COMMENT] For the conclusion section from page 16 of 22 to 17 of 22, it would help for the Author(s) to create a subheading on the implications of this general overview. The reader(s) would be interested to know the implications from this general overview. Is it a managerial implication or is it a practical implication?

 [OUR RESPONSE] This is a good idea, thanks. We do not have enough to write an entire section; however, we added this sentence to the Conclusion and hope it clarifies that this is a practical application “Combining our practical experience as both scholars and regular users of Gen AI with a broad range of informational sources this semi-formal text has practical implications in helping those in academia – students, faculty and staff – to be informed and improve their work.”

***Comment No. 3***

 [COMMENT] It is very good that the first sentence of the abstract mentions the review is for academics, students, workers, researchers and teachers. However, in the conclusion section there is no information in reference to students. There is a bit of mismatch here and no consistency. The Author(s) are advised to show what this review has discussed in reference to students as well as show it in the conclusion section.

 [OUR RESPONSE] We agree and already the new sentence in the Conclusion (mentioned in the previous response) mentions students directly, and we added the following sentence “This is particularly relevant for students, as they use Gen AI at the highest rates, are the least skeptical of its risks and in a phase of critical thinking development which AI might blunt”

  [HN4]To be edited at the end

  [HN5]fixed

  [HN6]fixed

  [HN7]fixed

  [HN8]Do we cite the models or? I’m not sure what kind of citations they want.

  [HN9]

[HN10]To be edited at the end
